# Impact of ERAP1 downregulation on the pathogenesis of DSS-induced colitis and therapeutic response to sulfasalazine

**DOI:** 10.3389/fimmu.2025.1645678

**Published:** 2025-09-05

**Authors:** Bushra Riaz, Hye-Myung Ryu, S. M. Shamsul Islam, Rahar Babita, Je Kyung Seong, Ho Lee, Eunjoo H. Lee, Seonghyang Sohn

**Affiliations:** ^1^ Department of Biomedical Sciences, Graduate School of Ajou University, Suwon, Republic of Korea; ^2^ Brain Korea 21 R&E Initiative for Advanced Precision Medicine, Ajou University School of Medicine, Suwon, Republic of Korea; ^3^ Department of Microbiology, Ajou University School of Medicine, Suwon, Republic of Korea; ^4^ Laboratory of Developmental Biology and Genomics, Research Institute for Veterinary Science, Seoul National University, Seoul, Republic of Korea; ^5^ Brain Korea 21 Plus Program FOUR Future Veterinary Medicine Leading Education and Research Center, College of Veterinary Medicine, Seoul National University, Seoul, Republic of Korea; ^6^ Korea Model Animal Priority Center (KMPC), Seoul National University, Seoul, Republic of Korea; ^7^ Graduate School of Cancer Science and Policy, National Cancer Center, Goyang, Republic of Korea; ^8^ Department of East-West Medical Science, Kyung Hee University, Yongin, Republic of Korea

**Keywords:** endoplasmic reticulum aminopeptidase-1, DSS-induced colitis, sulfasalazine, immune response, mouse model

## Abstract

**Introduction:**

Ulcerative colitis (UC) is a life-threatening heterogeneous condition characterized by inflammation of the colon. Endoplasmic reticulum aminopeptidase 1 (ERAP1) is essential for antigen processing and immune regulation, however, its specific role in UC pathogenesis and therapeutic response remains unclear. This study aimed to investigate the role of ERAP1 in the response to sulfasalazine, a standard treatment for UC, using an ERAP1-heterozygous (ERAP1^+/-^) mouse model susceptible to colitis.

**Methods:**

Wild-type (WT) and ERAP1^+/-^ mice were treated with 2.5% dextran sulfate sodium to induce colitis, followed by sulfasalazine administration. Colitis severity was assessed through histopathology. Immune cell populations, including neutrophils, dendritic cells, T cells, and NK1.1+ cells, were analyzed using flow cytometry. RNA sequencing of colonic tissues was performed to assess gene expression changes associated with reduced ERAP1 expression.

**Results:**

ERAP1^+/-^ mice exhibited mildly increased susceptibility to DSS-induced colitis, with greater weight loss and distinct alterations in immune cell infiltration compared to WT mice. These differences were further pronounced after sulfasalazine treatment. RNA sequencing identified 428 differentially expressed genes between ERAP1⁺/⁻ and WT mice. Among these, 28 genes were previously associated with colitis or colorectal cancer, of which 11 were upregulated and 17 downregulated in ERAP1^+/-^ mice. RT-qPCR confirmed significantly elevated expression of *Anxa9, Atp2a1, and Hepacam2* in ERAP1^+/-^ mice after sulfasalazine treatment, indicating a differential therapeutic response.

**Conclusion:**

Collectively, our findings show that partial ERAP1 deficiency promotes immune dysregulation, alters the expression of inflammation-associated genes, and impairs sulfasalazine efficacy. Therefore, ERAP1 may serve as a key regulator in the pathogenesis of UC and a potential target for therapy.

## 1 Introduction

Ulcerative colitis (UC) is a type of inflammatory bowel disease (IBD) with a multifaceted etiology. It primarily manifests as mucosal inflammation, commonly involving the rectum and extending proximally, with potential progression to encompass the entire colon (1, 2). Clinically, UC is characterized by bloody stools, abdominal cramping, weight loss, and rectal hemorrhage, while histological features include mucosal inflammation, crypt distortion, and crypt abscess formation (3). The chronic inflammatory environment observed in UC is a multifactorial process that is primarily defined by genetic predisposition, epigenetic modifications, the dynamic composition of the gut microbiota, and the complex responses of the host immune system (1, 4–6). A recent meta-analysis of genome-wide association studies (GWAS) identified over 200 IBD-associated loci related to UC and Crohn’s disease (CD). These loci contain genes involved in autophagy, epithelial barrier function, host defense mechanisms against pathogens, microbial recognition, innate and adaptive immune response, and cytokine signaling (7). However, the exact etiology of UC remains unclear, and the disease can eventually progress to colitis-associated colorectal cancer. This serious health issue places a significant burden on the healthcare system (8). Given the rising global prevalence of UC and its impact on quality of life, the discovery of novel biomarkers and therapeutic targets is crucial to improving disease management and patient outcomes.

The administration of dextran sulfate sodium is a commonly used chemical method to induce colitis by damaging the integrity of intestinal epithelial cells through its cytotoxic effects. This disruption of the epithelial barrier allows immune cells to encounter antigens such as microbes, thereby triggering a swift and intense inflammatory immune response (9). Several animal models of IBD have been developed to investigate the molecular pathways underlying colitis pathogenesis. Among these, DSS-induced colitis is a well-established and widely used model for studying intestinal inflammatory responses. It closely mimics the morphological and clinical features of human UC, including bloody stools, abdominal cramping, rectal hemorrhage, colon shortening, loss of goblet cells, and mucosal ulcerations (10, 11). Due to its simplicity and reproducibility, the DSS model allows for modulation of disease severity and duration by adjusting the dose and treatment schedule, making it suitable for modeling various phases of intestinal inflammation, including acute, relapsing, and chronic forms. Furthermore, it provides a valuable platform for examining tissue repair and persistent wound healing following epithelial injury (12). These characteristics make the DSS-induced colitis model an appropriate tool to study immune responses and chronic inflammation in the pathogenesis of UC.

The immune response in UC involves several key immune cells, including neutrophils, dendritic cells (DCs), T cells, and natural killer (NK) cells. When excessively activated, these immune cells contribute to disease progression by promoting inflammation and disrupting intestinal homeostasis (6). Neutrophils play a central role in driving intestinal inflammation in UC by secreting inflammatory mediators, such as reactive oxygen species (ROS), chemokines, and neutrophil extracellular traps (NETs). These factors compromise the epithelial barrier, increase intestinal permeability, and recruit additional immune cells, thereby amplifying inflammatory response (13). DCs, as key antigen-presenting cells, are essential for recognizing gut antigens and shaping adaptive immune responses. However, in patients with IBD, clinical evidence shows that DCs undergo significant alterations within inflamed mucosa, including changes in cell numbers, impaired tolerogenic function, and dysregulated cytokine production. These dysfunctions contribute to the breakdown of immune tolerance to the gut microbiota, leading to excessive polarization and activation of inflammatory T cells, which sustain chronic intestinal inflammation and accelerate disease progression (14, 15). T cells also play a critical role in UC pathology. Both CD4+ and CD8+ T cells infiltrate the intestinal mucosa and peripheral blood of IBD patients across all age groups. These cells exacerbate inflammation by releasing pro-inflammatory cytokines, disrupting epithelial barrier integrity, and promoting immune dysregulation (16, 17). Additionally, NK cells contribute to the chronic inflammatory environment of UC. Depletion of NK cells has been shown to worsen colitis by intensifying neutrophil-mediated inflammation, resulting in increased leukocyte infiltration, tissue damage in the colon, and heightened pro-inflammatory responses (18). Together, these immune cells orchestrate the complex inflammatory milieu observed in colitis, with their interactions critically influencing disease severity and outcomes (19, 20). The interplay between immune cells and related inflammatory mediators underscores the complexity of colitis pathogenesis and offers valuable insights into potential targets for immune-modulating therapies in UC.

Endoplasmic reticulum aminopeptidase 1 (ERAP1) plays a critical role in the functioning of the endoplasmic reticulum by catalyzing the removal of amino-terminal sequences from antigenic precursors. This process profoundly influences the number of epitopes presented on the cell surface, thereby modulating immune response to pathogens (21). Moreover, variations in ERAP1 due to single-nucleotide polymorphisms (SNPs) can alter its peptide-processing function, influencing the repertoire of antigens displayed and impacting CD8^+^ T cell responses. These changes may lead to immune dysregulation and increased susceptibility to inflammatory diseases (22–24). A previous study showed that ERAP1-deficient mice exhibit increased susceptibility to DSS-induced colitis, characterized by extensive ulceration and colonic irritation. Histological analysis of colonic tissues of DSS-treated ERAP1^−/−^ mice showed severe inflammation and clusters of proliferating lymphocytes, indicating elevated immune activation (25, 26). These findings highlight the crucial role of ERAP1 in immune regulation and disease susceptibility, although the precise molecular mechanisms driving increased colitis severity in the absence of ERAP1 remain unclear. Sulfasalazine, a disease-modifying antirheumatic drug (DMARD), is commonly used to manage inflammatory conditions, such as rheumatoid arthritis and UC (27, 28). It is a prodrug composed of 5-aminosalicylic acid (5-ASA) and sulfapyridine (SP), linked via an azo bond that prevents absorption in the upper gastrointestinal tract (29). Upon reaching the colon, bacterial enzymes such as azoreductases cleave this bond, releasing 5-ASA and SP locally (30). While SP is efficiently absorbed and can contribute to systemic side effects, 5-ASA remains primarily within the colon, promoting mucosal repair (31, 32). Although 5-ASA plays a role in reducing UC symptoms, it is important to note that the therapeutic efficacy of sulfasalazine is largely attributed to the intact compound rather than 5-ASA alone (33). However, its precise mechanism of action remains incompletely understood. Therefore, investigating the effects of sulfasalazine in DSS-induced colitis models with partial ERAP1 expression may provide valuable insights into colitis pathogenesis and therapeutic modulation.

Recent studies have highlighted the role of ERAP1 in the progression of colitis and response to sulfasalazine, using a colitis-susceptible ERAP1 heterozygous (ERAP1^+^/^−^) mouse model. Notably, despite the known anti-inflammatory properties of sulfasalazine, its administration failed to alleviate colitis symptoms in ERAP1^+^/^−^ mice. Instead, it exacerbated disease severity by modulating the activation markers expressed on neutrophils, DCs, T cells, and NK cells, as well as altering the expression profiles of inflammation-related genes. These findings highlight the critical role of ERAP1 in maintaining intestinal immune homeostasis and suggest that its deficiency may impair the effectiveness of conventional anti-inflammatory treatments. Elucidating the mechanistic basis of ERAP1 deficiency in colitis pathogenesis could provide novel insights for developing therapeutic strategies for IBD.

## 2 Materials and methods

### 2.1 Generation of ERAP1 heterozygous (ERAP1^+^/^−^) mice

ERAP1^+/tm1a^ mice were generated by the Korea Mouse Phenotyping Center (KMPC) using a clone of mouse embryonic stem cells carrying a mutant ERAP1 allele (ERAP1^tm1a (EUCOMM)Wtsi^), as previously described (34). To obtain ERAP1^+^/^−^ mice, ERAP1^+/tm1a^ were crossed with a Cre recombinase-expressing strain, and the offspring were maintained on a C57BL/6 background. For the generation of ERAP1 knockout mice, a targeting vector was constructed with two loxP sites flanking exons 6 and 7 of the ERAP1 gene, enabling the specific deletion of these exons upon Cre recombinase expression.

Genotyping was performed using the tail tissue collected approximately two weeks after birth. Genomic DNA was extracted from tail tissue using the DNeasy Blood & Tissue kit (Qiagen, Hilden, Germany) according to the manufacturer’s instructions. The following primer sets were used for genotyping: ERAP1-F 5′- GTG GTC ATT AGC AGG AGG CA - 3′; neoF, 5′- GGG ATC TCA TGC TGG AGT TCT TCG - 3′, and ERAP1-ttR 5′-CTC TCT GTA TGT GGT CAG TCC C - 3′. The amplicon (bp) of the PCR products for the mutant alleles and wild-type (WT) were 959 and 808, respectively. PCR protocol included the following steps: 95 °C for 5 minutes (min); 40 cycles of 95 °C for 1 min, 60 °C for 1 min, and 72 °C for 1 min, this was finally followed by 72 °C for 7 min.

### 2.2 Animal management

Germ-free ERAP1 WT and ERAP1^+^/^−^ mice (8 weeks old, 20 – 22 g) were used and housed in a specific pathogen-free (SPF) facility. All mice were on the C57BL/6 background. Environmental conditions were controlled: a 12-hour (h) light/dark cycle, a stable temperature of 22 °C, and a relative humidity of 22 – 30%. Mice had access to autoclaved water and food at all times. Mouse cages were changed weekly. Approval for animal work was carefully assessed following the guidelines set by the Ajou University Animal Care and Use Committee (IACUC - 2024 - 0003). Mice were continuously observed and monitored throughout the entire experiment.

### 2.3 Study design

Eight-week-old ERAP1 WT and ERAP1^+^/^−^ mice were assigned randomly to three groups each: WT, WT + DSS, WT + DSS + SSZ; and ERAP1^+^/^−^, ERAP1^+^/^−^ + DSS, ERAP1^+^/^−^ + DSS + SSZ. Each group consists of 4 to 6 mice. Group 1 served as the control and received only regular water for 28 days. Group 2 underwent a cyclic DSS treatment to induce colitis. Mice were administered 2.5% DSS (molecular weight: 36 kDa-50 kDa; MP Biomedicals, LLC, Canada) in their drinking water for 7 days, followed by 7 days of recovery with regular water. This cycle was repeated once more (7 days of 2.5% DSS followed by 7 days of regular water), resulting in a 28-day experimental period. This alternating DSS–recovery regimen was chosen based on previous studies to induce moderate, non-lethal colitis in C57BL/6J mice (35). It more closely replicates the episodic flare-recovery pattern of human ulcerative colitis (36) and provides a relevant model for studying chronic intestinal inflammation. Group 3 followed the same cyclic DSS protocol as Group 2; however, during the final 7-day recovery phase, sulfasalazine (SSZ, 40 mg/kg, Sigma, USA) was added to the drinking water to assess its therapeutic efficacy. On day 29, mice were euthanized via CO_2_ inhalation in accordance with IACUC guidelines. CO_2_ was introduced at a rate of 10%–30% of the chamber volume per minute until loss of consciousness, after which death was confirmed by the cessation of respiration and heartbeat. Following anesthesia, euthanasia was completed by cervical dislocation. Colon, spleen, and peripheral blood samples were then collected for further analysis.

### 2.4 Sample collection

Peripheral blood leukocytes (PBLs), spleen, and intestinal epithelial cells (IELs) were isolated from each mouse after euthanasia. PBLs were collected by cardiac puncture. The whole spleen was carefully excised and gently mashed using a 70 µm filter to prepare a single-cell suspension. PBLs and spleen tissues were treated with ammonium-chloride-potassium buffer to lyse the red blood cells. IELs were collected using a procedure described previously (37). In brief, after euthanasia, the colon was carefully removed using scissors. Fat, connective tissue, and visible Peyer’s patches were surgically removed before further processing to ensure the purity of the IEL population. The intestines were then thoroughly rinsed three times using ice-cold phosphate-buffered saline (PBS) to eliminate gut contents. After longitudinal incision along the intestine, they were further sliced into 0.5 cm pieces and transferred into 50 mL tubes containing RPMI media enriched with 2% FBS, 5% DTT, and 0.5M EDTA. The samples were incubated at 37 °C for 1h and shaken vigorously every 15 seconds (s) to detach the epithelial cells. After repeating this process once, the cell suspension was filtered through a 70 µm filter to remove debris. Finally, the epithelial cells were collected by centrifugation at 3000 rpm for 5 min at 4 °C. The cell suspensions obtained from PBLs and IELs were washed and resuspended in appropriate buffers and used for subsequent analyses such as flow cytometry.

### 2.5 Flow cytometry

The frequencies of neutrophils, DCs, and lymphocytes in PBLs and IELs were analyzed using flow cytometry as previously described (34). Cell suspensions from all collected tissues were stained with fluorescently labeled antibodies targeting specific cell surface markers. The concentration of immune cells was adjusted to 1 × 10^6^ cells per sample and then incubated with the antibody mixture for 30 min on ice in the dark. After staining, cells were washed with PBS and resuspended in the appropriate buffer. Data acquisition and analysis were performed using Cytek Aurora flow cytometer (Cytek Biosciences, Fremont, CA, USA), identifying immune cell populations based on surface marker expressions. Anti-mouse antibodies used in this study are provided in **Supplementary Table S1** and all purchased from eBioscience (San Diego, CA, USA). Neutrophils, NK cells, and CD8^+^ T cells were identified by flow cytometry using standard gating strategies, as depicted in **Supplementary Figure S1**.

### 2.6 Colonic histopathology

Colonic tissues were collected from mice, washed with cold PBS, and fixed in 4% paraformaldehyde solution at 4 °C for preservation. The fixed tissues were dehydrated through a graded series of alcohols and embedded in paraffin blocks for histopathological analysis. Paraffin-embedded tissue blocks were sectioned into 4 µm thick slices using a microtome (Reichert-Jung Biocut 2030, Ramsey, MN, USA). The tissue sections were deparaffinized in xylene, rehydrated through a graded ethanol series, and stained with hematoxylin and eosin (H&E) for the evaluation of histopathological changes under a light microscope. Histological inflammation was assessed using the simplified Geboes score as previously described (38). This scoring system comprises five categories: grade 0, structural changes without active inflammation; grade 1, chronic inflammatory infiltrates in the lamina propria; grade 2, presence of neutrophils in the lamina propria and/or epithelium; grade 3, epithelial damage with crypt destruction or erosion; and grade 4, ulceration. Higher grades correspond to greater histological disease activity.

### 2.7 RNA-sequencing

Total RNA was extracted from the intestine tissue using Trizol reagent (Invitrogen). The quality of RNA was evaluated by Agilent 4200 TapeStation System (Agilent Technologies, Santa Clara, CA, USA), and RNA integrity was confirmed by agarose gel electrophoresis. RNA integrity number (RIN) was measured using the Agilent 2100 Bioanalyzer (Agilent Technologies, Palo Alto, CA, USA). Only RNA samples with a RIN score exceeding 7 or 8 were selected for further library preparation and sequencing. Equal amounts of RNA were pooled from three individual samples per group to reduce interindividual variability. Library preparation and RNA sequencing were conducted using the QuantSeq 3’ mRNA-Seq Library Prep Kit FWD (Lexogen, Vienna, Austria) according to the manufacturer’s instructions. Briefly, the process consisted of preparing RNA, hybridizing with an oligo-dT primer with an Illumina-compatible sequence at the 5’ end, and performing reverse transcription. After the RNA template was degraded, second-strand synthesis was initiated using random primers with Illumina-compatible linker sequences at the 5’ end. The generated double-stranded library was purified using magnetic beads to remove unintegrated reaction components. The library was then amplified using the entire adapter sequence required for cluster formation. The completed libraries were purified after amplification to eliminate any remaining PCR components. Single-end 75 bp sequencing was performed using the NextSeq 500 platform (Illumina, Inc., USA). Raw sequencing data were quality controlled using FastQC prior to data processing (39). Sequenced reads were trimmed for adapter sequences, and low-quality reads were filtered using bbduk (40). The purified reads were aligned to the reference genome using STAR (41), and read quantification was performed using HTSeq-count (42). Read counts were processed based on TMM+CPM.

### 2.8 Differentially expressed gene analysis

DEGs were identified in intestinal samples of ERAP1 WT and ERAP1^+^/^−^ mice. Sequencing reads were aligned to the GENCODE GRCm38 v23 transcript reference using Bowtie2, and gene expression levels were quantified using RSEM. Expression values were normalized using the trimmed mean of M values (TMM) method. DEG analysis was performed using the edgeR package. Genes with a false discovery rate (FDR)-adjusted P-value < 0.05 and an absolute log2 fold change (|logFC|) > 2 were considered differentially expressed. Genes with logFC < 0 were classified as downregulated, while those with logFC > 0 were classified as upregulated.

### 2.9 Gene expression analysis

To quantify gene expressions, real-time quantitative PCR (qRT-PCR) was conducted using the 7500 Real-Time PCR System (Applied Biosystems). Total RNA was isolated from spleen tissue using TRIzol reagent (Thermo Fisher, Waltham, MA, USA), and the concentration and purity were measured using a spectrophotometer. The extracted RNA was reverse-transcribed into cDNA using the PrimeScript cDNA Synthesis Kit (Takara, Japan) according to the manufacturer’s protocol. SYBR Green PCR Master Mix (Applied Biosystems, Foster City, CA, USA) was used for PCR amplification. Each qRT-PCR reaction was performed in a total volume of 20 µL, including 1 µL of synthesized cDNA template. The thermal cyclic program was set as follows: initial denaturation was performed at 94 °C for 2 min, followed by 40 cycles of denaturation at 94 °C for 3 s, annealing at 55 °C for 30 s, and extension at 72 °C for 30 s. The final extension step was performed at 72 °C for 10 min. Gene expression was normalized to β-actin as an internal control, and relative quantification was determined using the 2−ΔΔCt method. Specific primers for the target genes are presented in **Table 1**.

**Table 1:** Primer sequences for PCR.

Gene	Forward	Reverse
Anxa9	CACTTCAGCAGGCTGGAGAATC	CGGATAGCATCCTCCAGTTCCT
Atp2a1	TCATTGCCAACGCCATTGTG	CAGCCCGATAGACCTTTCCC
Hepacam2	GAAGCATGGTTGGGCTCTCT	TGGACAGTGTATGACGGCAC
β-actin	TGTCCACCTTCCAGCAGATGT	AGCTCAGTAACAGTCCGCCTAG

### 2.10 Gene Ontology and Kyoto Encyclopedia of Genes and Genomes pathway enrichment analyses

GO analysis is a widely used method for determining the biological functions of genes and their RNA or protein products in high-throughput transcriptomic or genomic datasets (43). KEGG pathway enrichment analysis provides insights into molecular interaction and reaction networks by integrating information on genomes, biological pathways, diseases, drugs, and chemicals. The Database for Annotation, Visualization, and Integrated Discovery (DAVID; version 6.7; https://davidbioinformatics.nih.gov/) was used to perform functional annotation and enrichment analysis. DAVID offers a comprehensive suite of GO term annotations and KEGG pathway enrichment to help interpret the biological significance of large gene lists (44). Functional categories and pathways were considered statistically significant if the p-value was less than 0.05.

### 2.11 Statistical analysis

To assess significant differences among experimental groups, a one-way analysis of variance with Tukey’s *post hoc* test and a two-tailed t-test (95% confidence interval) were performed, and appropriate corrections were applied for multiple comparisons. All statistical analyses were performed using GraphPad Prism version 8.3.1 (GraphPad Software, La Jolla, CA, USA). Each experiment was conducted at least twice, and the data were expressed as the mean ± standard deviation. The results were considered significant at the significance level of p < 0.05.

## 3 Results

### 3.1 Impact of ERAP1 on colitis severity and sulfasalazine efficacy in DSS-treated mice

**Figure 1:**
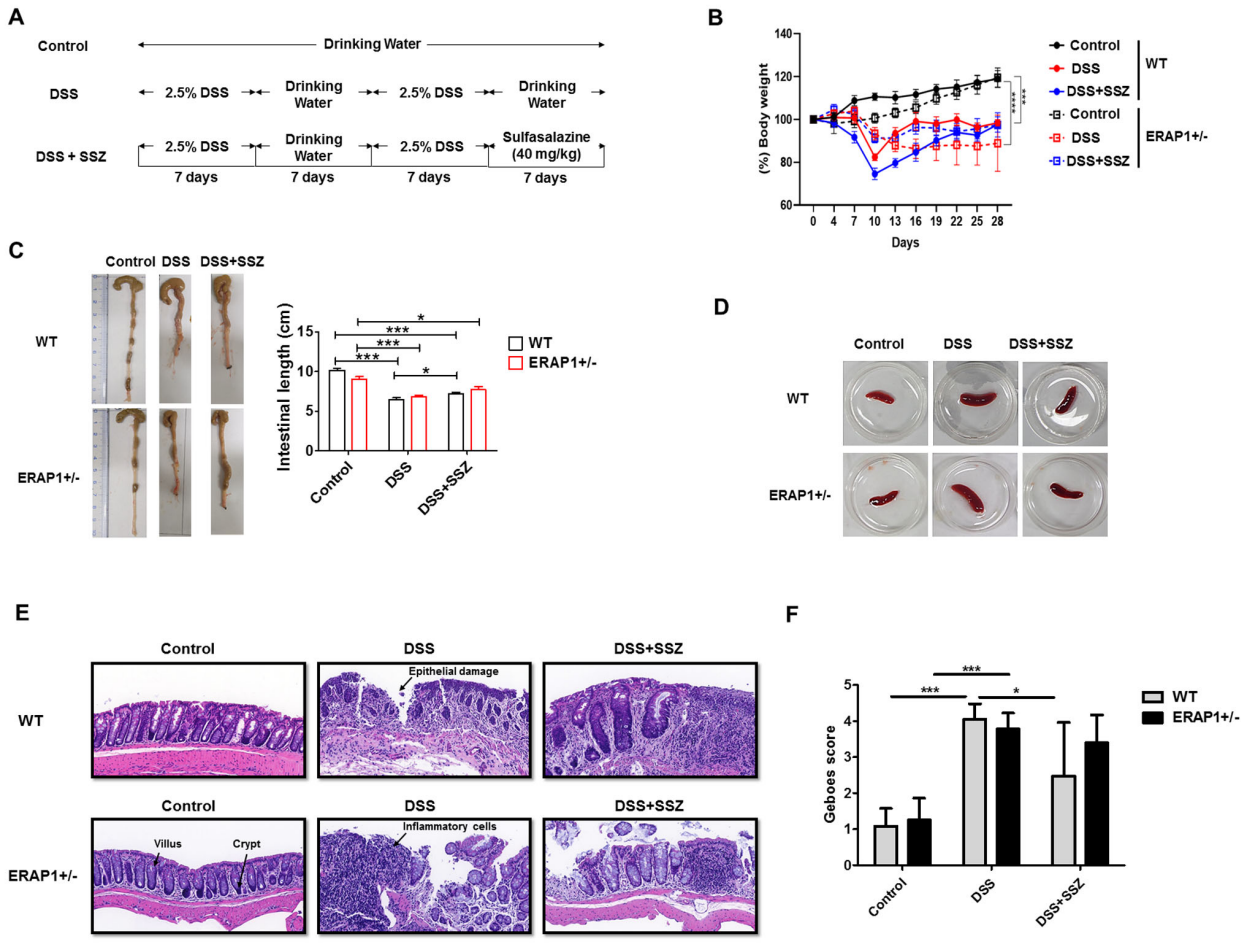
Role of ERAP1 in modulating colitis severity and sulfasalazine efficacy in DSS-induced colitis. **(A)** Schematic timeline of DSS administration and sulfasalazine treatment in ERAP1 WT and ERAP1^+^/^−^ mice (n = 4 – 6 per group). **(B)** Body weight changes were monitored throughout the experimental period. **(C)** Colon lengths were measured at the end of the experiment as an indicator of colitis severity. **(D)** Representative images of spleens from each group. **(E)** Representative H&E-stained colon sections from control, DSS-treated, and sulfasalazine-treated ERAP1 WT and ERAP1^+^/^−^ mice, showing histopathological alterations in colonic architecture. **(F)** Representative H&E-stained colonic sections from each group were evaluated, and histological damage was quantified using the simplified Geboes scoring system. A total of six samples (n = 6) per group were analyzed. Data are expressed as means ± SD. Statistical significance is indicated as *p < 0.05, ***p < 0.001, and p < 0.0001.

DSS-induced colitis model was used to investigate the role of ERAP1 in colitis development. ERAP1 wild-type (WT) and ERAP1^+^/^−^ mice were given 2.5% DSS in their drinking water for two separate 7-day cycles, each followed by a 7-day recovery period with plain water, over a total duration of four weeks. During the final 7 days of the second DSS cycle, the treatment group received sulfasalazine in place of plain water (**Figure 1A**). Body weight was monitored every three days throughout the study. Mice treated with DSS exhibited significant weight loss, with WT mice losing approximately 20% and ERAP1^+^/^−^ mice losing around 30% of their initial body weight. Sulfasalazine treatment did not restore body weight to baseline levels (**Figure 1B**). Colon length, a key indicator of colitis severity, was significantly reduced in DSS-treated WT and ERAP1^+^/^−^ mice compared to controls. While sulfasalazine treatment restored colon length in DSS-induced WT mice, it had no significant effect in DSS-treated ERAP1^+^/^−^ mice (**Figure 1C**). Additionally, sulfasalazine treatment led to a reduction in spleen size in both WT and ERAP1^+^/^−^ colitis mice compared to those treated with DSS alone (**Figure 1D**). To further assess the impact of ERAP1 on colonic morphology during inflammation, H&E staining was performed. Control mice exhibited well-preserved colonic architecture characterized by intact crypts, organized villous structures, and no signs of ulceration or inflammatory infiltration. In contrast, DSS-treated WT and ERAP1^+^/^−^ mice showed severe colonic damage, including structural disorganization, villous atrophy, extensive inflammatory cell infiltration, epithelial injury, and increased apoptosis. Sulfasalazine treatment ameliorated some of these pathological alterations in WT colitis mice but failed to improve colonic damage in ERAP1^+^/^−^ mice (**Figure 1E**). Histological evaluation using the Geboes scoring system showed that both WT and ERAP1^+^/^−^ mice treated with DSS had significantly higher scores than the control group, indicating severe colonic inflammation (*p* < 0.001). In WT mice, sulfasalazine treatment significantly reduced Geboes scores compared with the DSS group (*p* < 0.05), indicating partial improvement in mucosal damage. In contrast, sulfasalazine had no effect in ERAP1^+^/^−^ mice (**Figure 1F**), suggesting that partial loss of ERAP1 may compromise key mechanisms involved in colonic protection and the resolution of inflammation.

### 3.2 Changes in the frequencies of Ly6G+ CD11b+ neutrophils in ERAP1 WT and ERAP1^+^/^−^ mice during sulfasalazine treatment in colitis

**Figure 2:**
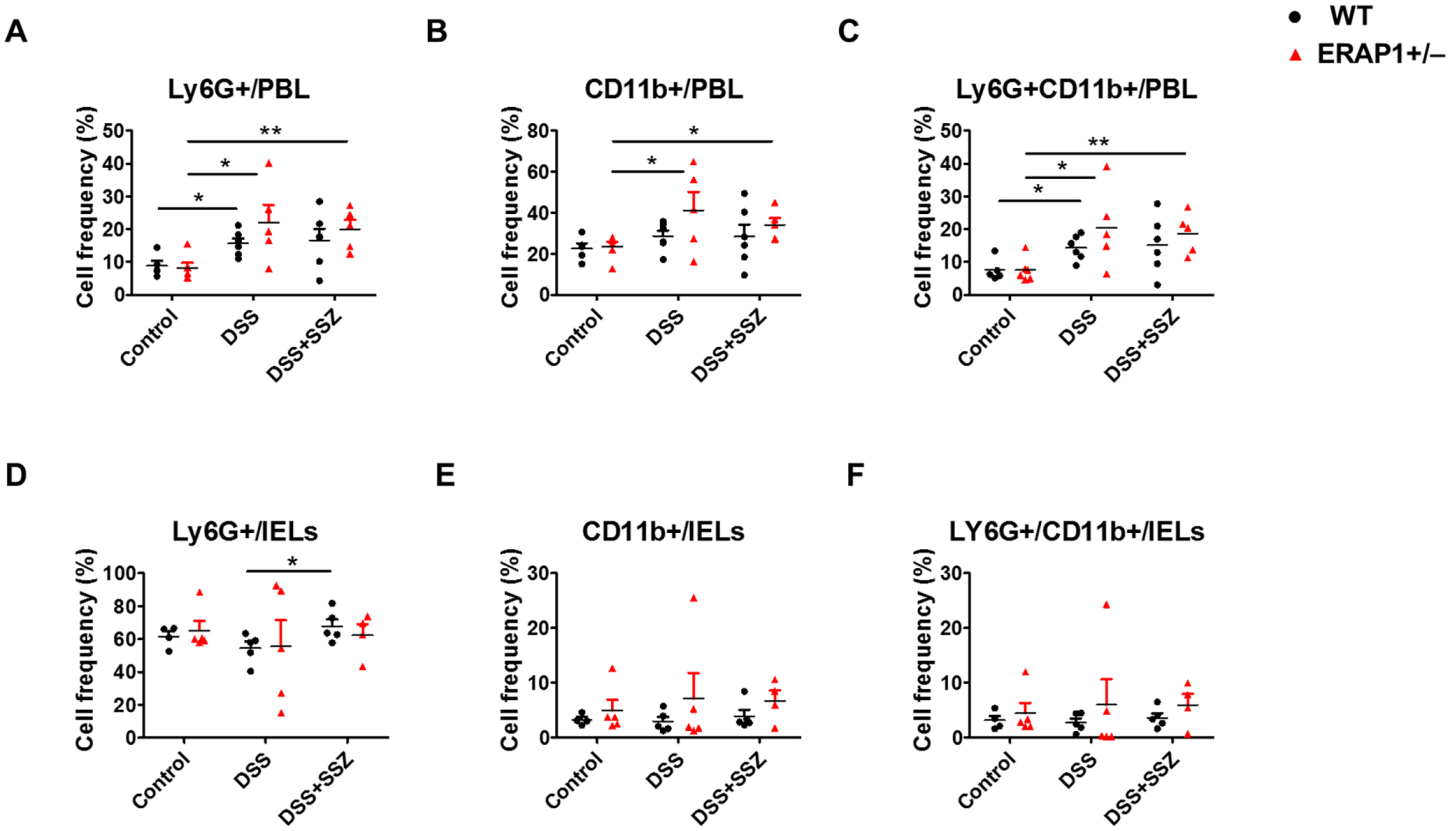
Effect of ERAP1 on neutrophil accumulation in ERAP1 WT and ERAP1^+^/^−^ mice with DSS-induced colitis and sulfasalazine treatment. The percentages of Ly6G+, CD11b+, and Ly6G+CD11b+ cells were assessed by flow cytometry in PBLs **(A-C)** and IELs **(D-F)** from 8-week-old ERAP1 WT and ERAP1^+^/^−^ mice. The mice were divided into three groups: controls, DSS-induced colitis, and DSS-induced colitis treated with sulfasalazine (40 mg/kg) Each group included 4 – 6 mice. Experiments were independently repeated at least three times. Data are expressed as the mean ± SD. *p < 0.05, **p < 0.01.

To assess the impact of ERAP1 on neutrophil infiltration during colitis progression, Ly6G+ CD11b+ neutrophils were analyzed in peripheral blood leukocytes (PBLs) and intraepithelial lymphocytes (IELs) of DSS-treated ERAP1 WT and ERAP1^+^/^−^ mice using flow cytometry. In PBLs, ERAP1^+^/^−^ colitis mice exhibited a significant increase in the frequencies of Ly6G+, CD11b+, and Ly6G+CD11b+ cells compared to ERAP1^+^/^−^ healthy controls (*p* < 0.05) (**Figures 2A-C**). Similarly, these neutrophil subsets were significantly elevated in sulfasalazine-treated ERAP1^+^/^−^ colitis mice compared to ERAP1^+^/^−^ controls (Ly6G+, *p* < 0.01; CD11b+, *p* < 0.05; Ly6G+CD11b+, *p* < 0.01), indicating that sulfasalazine treatment failed to suppress neutrophilic inflammation under ERAP1 haploinsufficient conditions. Moreover, Ly6G+ and Ly6G+CD11b+ cells were markedly elevated in WT colitis mice compared to WT healthy controls (*p* < 0.05) (**Figures 2A, C**). In IELs, Ly6G+ neutrophils were significantly increased in WT colitis mice treated with sulfasalazine compared to untreated controls (*p* < 0.05) (**Figure 2D**). However, the frequencies of CD11b+ and Ly6G+CD11b+ cells in IELs remained unchanged across all experimental groups (**Figures 2E, F**). Moreover, ERAP1^+^/^−^ mice exhibited a 70% increase in Ly6G+CD11b+ neutrophil levels compared to WT mice under colitis conditions, indicating that partial ERAP1 deficiency affects neutrophil infiltration in this model. These findings indicate that ERAP1 may play a role in regulating neutrophil accumulation during colitis.

### 3.3 ERAP1 modulates the co-stimulatory molecules of DCs in ERAP1 WT and ERAP1^+^/^−^ mice under colitis and sulfasalazine treatment

**Figure 3:**
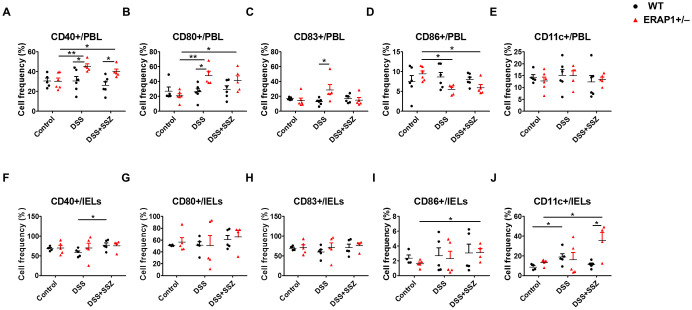
Modulation of DCs expressing co-stimulatory molecules in DSS-induced colitis with partial ERAP1 deficiency and sulfasalazine treatment. The percentages of CD40+, CD80+, CD83+, CD86+, and CD11c+ cells in PBLs **(A-E)** and IELs **(F-J)** were assessed using flow cytometry in 8-week-old ERAP1 WT and ERAP1^+^/^−^ mice. Experimental groups included controls, DSS-induced colitis mice, and colitis mice subjected to sulfasalazine (40 mg/kg). Each group consisted of 4 – 6 mice. Experiments were independently performed a minimum of three times. Data are presented as mean ± SD. *p < 0.05, **p < 0.01.

To observe the effect of ERAP1 on DC activation during colitis conditions, the expression of co-stimulatory molecules CD40, CD83, CD80, CD86, and CD11c was examined in PBLs and IELs of ERAP1 WT and ERAP1^+^/^−^ mice using flow cytometry. In PBLs, the frequencies of CD40+ and CD80+ cells were significantly increased in DSS-treated ERAP1^+^/^−^ mice (*p* < 0.01) and in sulfasalazine-treated ERAP1^+^/^−^ colitis mice (*p* < 0.05) compared to ERAP1^+^/^−^ healthy controls (**Figures 3A, B**). Additionally, DSS-treated ERAP1^+^/^−^ mice showed significantly higher frequencies of CD40+, CD80+, and CD83+ cells compared to DSS-treated WT mice (*p* < 0.05) (**Figures 3A-C**). Notably, CD40^+^ cell frequencies remained elevated in ERAP1^+^/^−^ colitis mice even after sulfasalazine treatment, compared to WT colitis mice (*p* < 0.05). In contrast, CD86+ cells were significantly reduced in ERAP1^+^/^−^ colitis mice (*p* < 0.05); this reduction was also observed following sulfasalazine treatment (*p* < 0.05) (**Figure 3D**). These findings suggest an altered immune response and potential dysregulation of antigen presentation under conditions of the ERAP1 haploinsufficiency. CD11c+ cell frequencies in PBLs remained unchanged across all groups (**Figure 3E**). In IELs, sulfasalazine-treated WT colitis mice exhibited a significant increase in CD40^+^ cell frequencies compared to untreated WT colitis mice (*p* < 0.05) (**Figure 3F**). Moreover, sulfasalazine-treated ERAP1^+^/^−^ mice showed significantly elevated CD86+ cell (*p* < 0.05) and CD11c+ cell (*p* < 0.05) frequencies compared to ERAP1^+^/^−^ healthy controls (**Figures 3I, J**). Further, ERAP1^+^/^−^ colitis mice showed a higher frequency of CD11c+ cells than WT colitis mice following sulfasalazine treatment. In addition, CD11c+ expression was significantly elevated in DSS-treated WT mice compared to WT healthy controls (*p* < 0.05) but was decreased by 55% upon sulfasalazine treatment, indicating a partial recovery effect of sulfasalazine in modulating immune responses during colitis (**Figure 3J**). The percentages of CD80+ and CD83+ cells in IELs remained unchanged across all experimental groups (**Figures 3G, H**). These results collectively indicate that ERAP1 is essential in regulating DC activation during experimental colitis by modulating the expression of co-stimulatory molecules.

### 3.4 Alteration of T cell and NK cell frequencies in ERAP1 WT and ERAP1^+^/^−^ mice under colitis and sulfasalazine treatment

**Figure 4:**
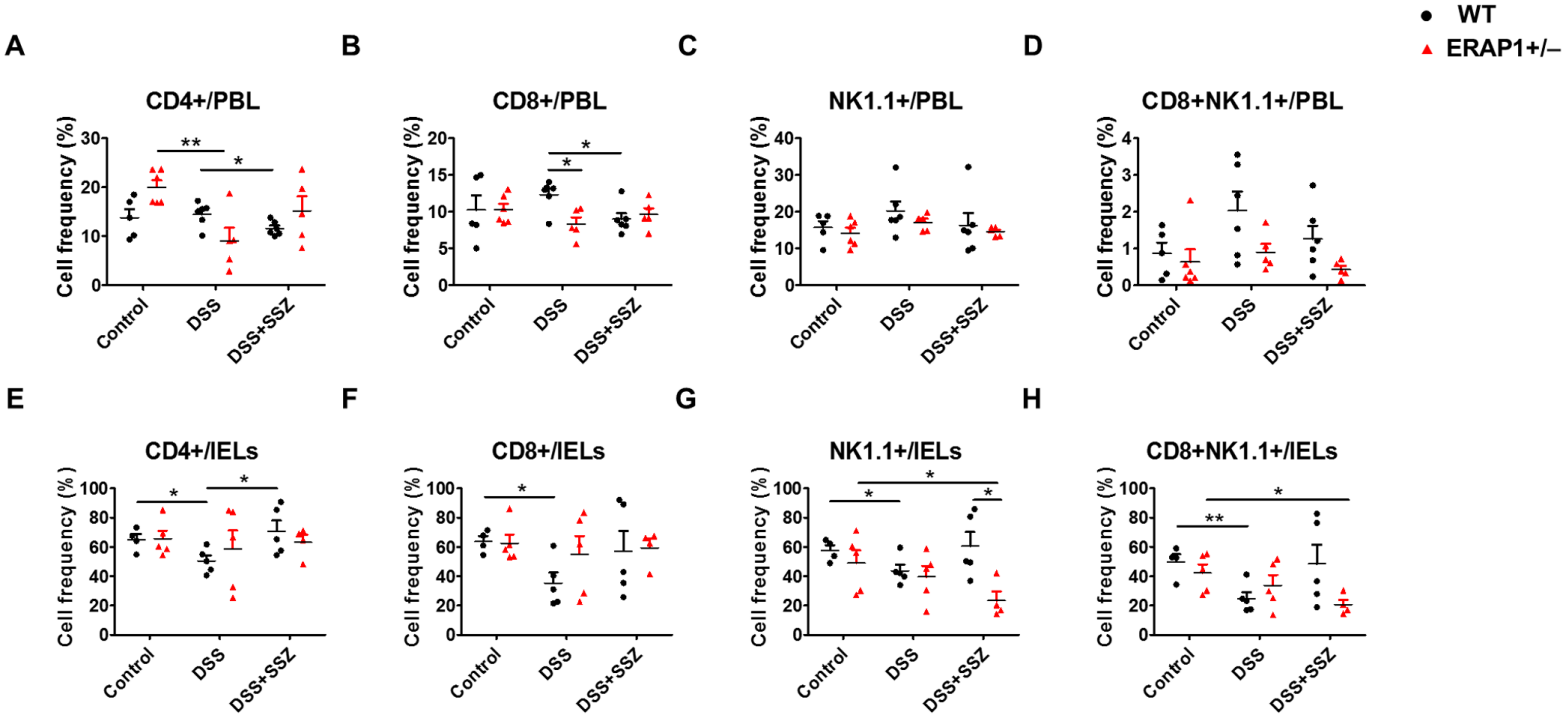
Effect of ERAP1 on T cell and NK cell frequencies in ERAP1 WT and ERAP1^+^/^−^ mice with DSS-induced colitis and sulfasalazine treatment. The percentages of CD4+, CD8+, NK1.1+, and CD8+NK1.1+ cells were assessed by flow cytometry in PBLs **(A-D)** and IELs **(E-H)** from 8-week-old ERAP1 WT and ERAP1^+^/^−^ mice. Experimental groups were divided into controls, DSS-induced colitis mice, and colitis mice subjected to sulfasalazine (40 mg/kg). Each group contained 4 – 6 mice. Experiments were independently performed at least three times. Data are expressed as the mean ± SD. *p < 0.05, **p < 0.01.

To elucidate the function of ERAP1 in immune response, the percentages of CD4+, CD8+, NK1.1+, and CD8+NK1.1+ cells in PBLs and IELs of ERAP1 WT and ERAP1^+^/^−^ mice were analyzed using flow cytometry. In PBLs, CD4+ T cells were significantly decreased in DSS-treated ERAP1^+^/^−^ mice than those from healthy ERAP1^+^/^−^ mice (*p* < 0.01) (**Figure 4A**). Similarly, CD4+ and CD8+ T cells were reduced after sulfasalazine treatment in WT colitis mice, whereas this was not the case in WT colitis mice (*p* < 0.05) (**Figures 4A, B**). Moreover, CD8+ T cells were considerably decreased in ERAP1^+^/^−^ colitis mice in contrast to WT colitis mice (*p* < 0.05) (**Figure 4B**). The percentages of NK1.1+ and CD8+NK1.1+ cells in PBLs remained unchanged across all experimental groups (**Figures 4C, D**). In IELs, WT colitis mice exhibited a significant reduction in CD4+, CD8+, NK1.1+, and CD8+NK1.1+ cells compared to WT control mice. However, sulfasalazine treatment led to an increase in these cell populations, with a significant rise in CD4^+^ T cells, indicating a potential immunoregulatory effect that promotes recovery during colitis (**Figures 4E-H**). NK cell depletion worsens colitis by promoting leukocyte infiltration, colonic damage, and pro-inflammatory profiles, as previously reported (18). In sulfasalazine-treated ERAP1^+^/^−^ colitis mice, the percentages of NK1.1+ (*p* < 0.05) and CD8+NK1.1+ (*p* < 0.05) cells were reduced than those in ERAP1^+^/^−^ healthy controls (**Figures 4G, H**). Additionally, NK1.1+ cell levels were markedly lower in ERAP1^+^/^−^ colitis mice compared to WT colitis mice (*p* < 0.05) after sulfasalazine treatment (**Figure 4G**). These results suggest that ERAP1 deficiency may impair immune responses, worsen colitis symptoms, and reduce the efficacy of sulfasalazine.

### 3.5 Differentially expressed genes under ERAP1 haploinsufficiency in the intestine tissue

**Figure 5:**
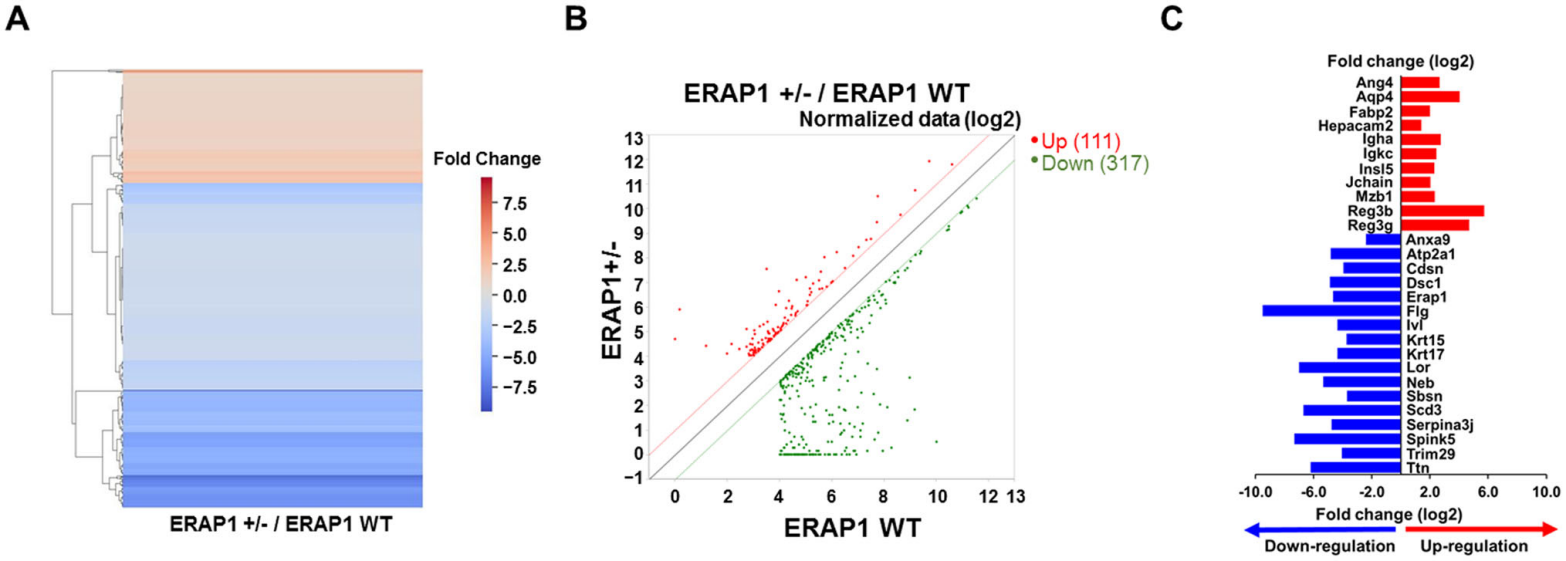
DEGs analysis in intestinal tissues of ERAP1 WT and ERAP1^+^/^−^ mice. **(A)** Heatmap of DEGs comparing ERAP1^+^/^−^ and ERAP1 WT mice. Upregulated genes are shown in red, and downregulated genes in blue. **(B)** Scatter plot representing DEGs between ERAP1^+^/^−^ and ERAP1 WT groups. Gene expression in ERAP1 WT and ERAP1^+^/^−^ mice is plotted on the x-axis and y-axis, respectively. Red dots indicate significantly upregulated genes (n = 111), while green dots represent significantly downregulated genes (n = 317). The closer the points are to the diagonals, the smaller the difference in representation between the two groups. **(C)** Representative bar graph of 28 genes with fold changes greater than 4 and p < 0.05. Red bars represent genes upregulated in ERAP1^+^/^−^ compared to ERAP1 WT, and blue bars represent genes downregulated.

RNA- sequencing analysis (RNA-seq) was performed to identify DEGs between ERAP1 WT and ERAP1^+^/^−^ mice. Total RNA was extracted from intestinal tissues of both groups for the analysis. As shown in the heatmap and scatter plot, there were 428 genes differentially expressed with fold change (FC) > 2 and p < 0.05. Among them, 111 genes (26%) were upregulated and 317 genes (74%) were downregulated in ERAP1^+^/^−^ samples compared to ERAP1 WT (**Figures 5A, B**).

Based on the RNA-seq results, 28 representative genes were selected based on a FC greater than 4 and p < 0.05, and reported relevance to colitis, colorectal cancer, or immune regulation, as well as their functional roles in inflammation, epithelial integrity, and immune cell modulation (45–47). These included *Ang4*, *Aqp4*, *Anxa9*, *Atp2a1*, *Cdsn*, *Dsc1*, *ERAP1*, *Fabp2*, *Flg*, *Hepacam2*, *Igha*, *Igk*c, *Insl5*, *Ivl*, *Jchain*, *Krt15*, *Krt17*, *Lor*, *Mzb1*, *Neb*, *Reg3b*, *Reg3g*, *Sbsn*, *Scd3*, *Serpina3j*, *Spink5*, *Trim29*, *Ttn*. Among them, 11 genes (*Ang4*, *Aqp4*, *Fabp2*, *Hepacam2*, *Igha*, I*gkc*, *Insl5*, *Jchain*, *Mzb1*, *Reg3b*, and *Reg3g*) were significantly upregulated in ERAP1^+^/^−^ mice, while 17 genes (*Anxa9*, *Atp2a1*, *Cdsn*, *Dsc1*, *ERAP1*, *Flg*, *Ivl*, *Krt15*, *Krt17*, *Lor*, *Neb*, *Sbsn*, *Scd3*, *Serpina3j*, *Spink5*, *Trim29*, *Ttn*) were downregulated relative to ERAP1 WT (**Figure 5C**).

### 3.6 Functional enrichment analysis

**Figure 6:**
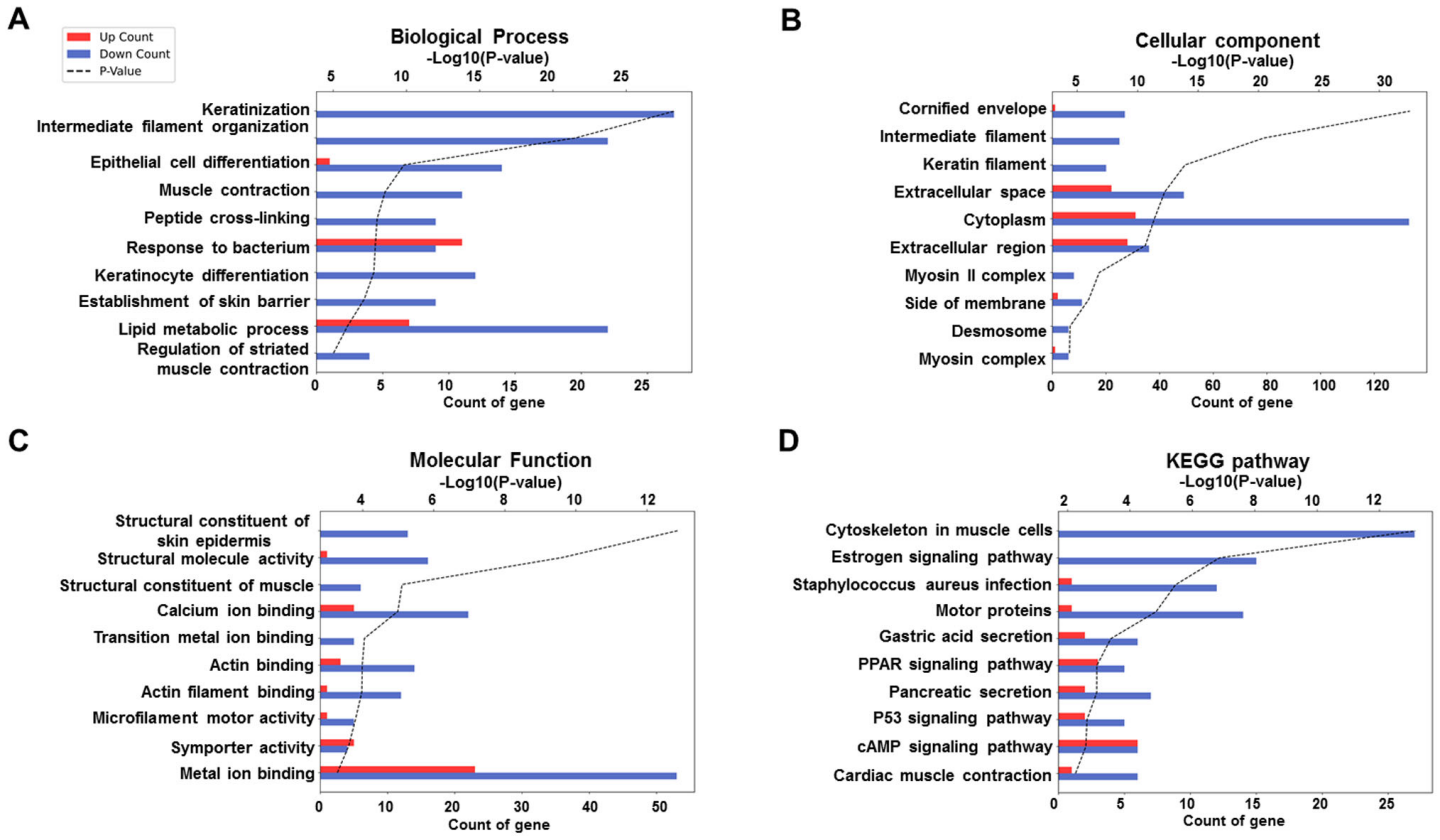
GO and KEGG enrichment analysis of DEGs between ERAP1 WT and ERAP1^+^/^−^ mice. **(A)** GO enrichment analysis in the category of Biological Process. **(B)** GO enrichment analysis in the category of Cellular Component. **(C)** GO enrichment analysis in the category of molecular function. **(D)** KEGG enrichment analysis. Bar graphs show the top 10 significantly enriched terms or pathways based on upregulated (red bars) and downregulated (blue bars) DEGs. The x-axis indicates the number of DEGs associated with each term (Count), and the black dotted line represents the statistical significance as -log10 (p-value).

To investigate the functional relevance of DEGs between ERAP1 WT and ERAP1^+^/^−^ mice, Gene Ontology (GO) and Kyoto Encyclopedia of Genes and Genomes (KEGG) pathway analyses were conducted. GO enrichment was categorized into three domains: biological process (BP), cellular component, and molecular function (MF). The top 10 significantly enriched GO terms for each category are presented in **Figure 6**. In the BP category, genes were significantly enriched in terms including keratinization, intermediate filament organization, epithelial cell differentiation, peptide cross-linking, keratinocyte differentiation, response to bacterium, establishment of skin barrier, lipid metabolic process, and regulation of striated muscle contraction (**Figure 6A**). For the CC category, enriched GO terms included cornified envelope, intermediate and keratin filaments, extracellular space, cytoplasm, extracellular region, myosin II complex, side of membrane, desmosome, and myosin complex (**Figure 6B**). In the MF category, genes were enriched in structural constituent of skin epidermis, structural molecule activity, structural constituent of muscle, calcium ion binding, transition metal ion binding, actin binding, microfilament motor activity, symporter activity, and metal ion binding (**Figure 6C**). KEGG pathway analysis revealed significant enrichment in pathways related to muscle contraction, estrogen signaling, Staphylococcus aureus infection, motor proteins, gastric acid secretion, peroxisome proliferator-activated receptor (PPAR) signaling, pancreatic secretion, p53 signaling, cAMP signaling, and cardiac muscle contraction (**Figure 6D**).

### 3.7 Validation of RNA-seq results and expression of immune regulatory markers using RT-qPCR

**Figure 7:**
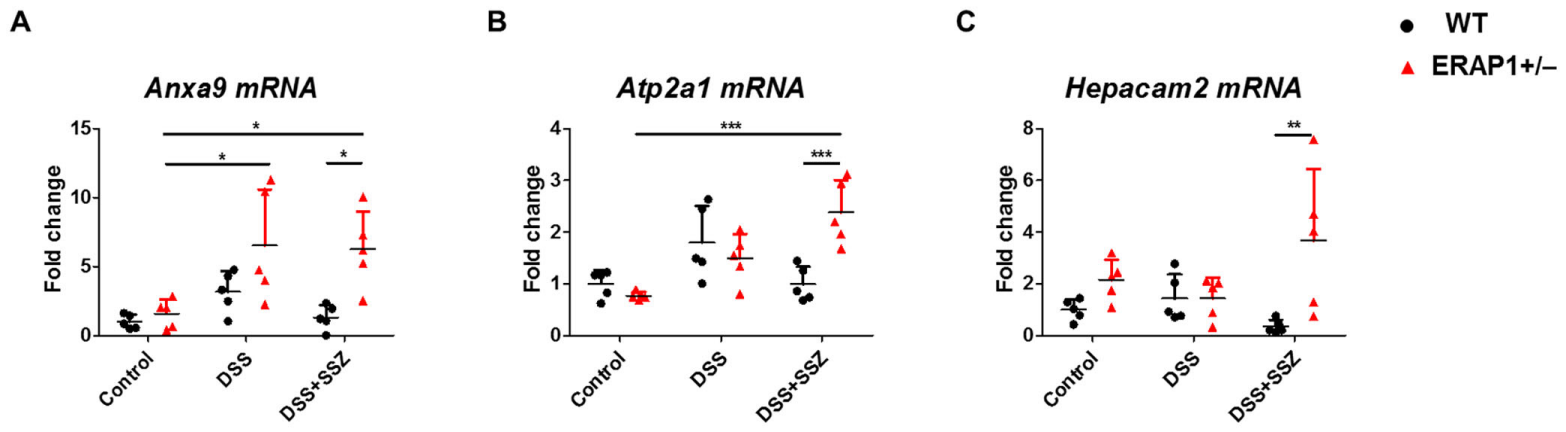
Validation of RNA-seq results by RT-qPCR. The relative mRNA expression levels of three selected transcripts, **(A)** *Anxa9*, **(B)** *Atp2a1*, and **(C)** *Hepacam2*, were validated by RT-qPCR using spleen tissue from ERAP1 WT and ERAP1^+^/^−^ mice. Mice were divided into three groups: controls, DSS-induced colitis mice, and colitis mice treated with sulfasalazine (40 mg/kg). Each group included 4 – 6 mice. All experiments were independently repeated at least three times. Data are presented as mean ± SD. *p < 0.05, **p < 0.01, ***p < 0.001.

To further evaluate the impact of ERAP1 expression on DSS-induced colitis and sulfasalazine treatment in ERAP1 WT and ERAP1^+^/^−^ mice, three genes (*Anxa9*, *Atp2a1*, and *Hepacam2*) were selected from the RNA-seq data and validated using RT-qPCR in spleen tissue. This approach allowed us to assess systemic immune responses in addition to local colonic changes. *Anxa9*, a novel marker associated with poor prognosis, was significantly upregulated in ERAP1^+^/^−^ colitis mice compared to ERAP1^+^/^−^ controls (*p* < 0.05), with a further significant increase observed following sulfasalazine treatment (*p* < 0.05) (**Figure 7A**). *Atp2a1*, a gene associated with increased risk of IBD (48), also showed a significant increase in expression in sulfasalazine-treated ERAP1^+^/^−^ colitis mice compared to ERAP1^+^/^−^ controls (*p* < 0.001) (**Figure 7B**). *Hepacam2*, a potential biomarker for distinguishing UC (49), was upregulated by 53% in ERAP1^+^/^−^ mice relative to ERAP1 WT mice in the control group, consistent with RNA-seq results (**Figure 7C**). Notably, mRNA expression levels of *Anxa9* (*p* < 0.05), *Atp2a1* (*p* < 0.001), and *Hepacam2* (*p* < 0.01) were all significantly upregulated in ERAP1^+^/^−^ colitis mice relative to WT colitis mice after sulfasalazine treatment (**Figures 7A-C**), indicating its potential role in colitis pathogenesis and therapeutic response. Additionally, three other DEGs (*Gadd45b*, *Reg3β*, *Spink5*) were examined for their reported relevance to IBD (50, 51), but none showed significant expression differences between groups (**Supplementary Figure S2**). Beyond RNA-seq validation, we also assessed Foxp3 and IL - 17, two immune regulatory markers associated with Treg and Th17 responses, respectively. RT-qPCR analysis of splenic tissue revealed a modest reduction in Foxp3 expression in ERAP1^+^/^−^ colitis mice compared with ERAP1^+^/^−^ controls (*p* < 0.05), which persisted even after sulfasalazine treatment. Furthermore, under colitis conditions, ERAP1^+^/^−^ mice exhibited a 64% decrease in Foxp3 expression compared with WT mice. In contrast, IL - 17 levels remained unchanged across all experimental groups (**Supplementary Figure S3**).

These results highlight changes in ERAP1 expression and differential regulation of these genes in response to sulfasalazine treatment, suggesting their potential as biomarkers for colitis progression and therapeutic efficacy.

## 4 Discussion

This study elucidates the role of ERAP1 in modulating immune responses during DSS-induced colitis and its impact on sulfasalazine efficacy. ERAP1^+^/^−^ mice on a C57BL/6 background were generated to assess the effects of partial ERAP1 expression on colitis susceptibility. ERAP1^+^/^−^ mice showed mildly increased susceptibility to DSS-induced colitis, evidenced by greater weight loss and altered expression of immune cell markers, including those of neutrophils, DCs, T cells, and NK cells even after sulfasalazine treatment.

Sulfasalazine, a first-line anti-inflammatory agent in UC (52), was less effective in ERAP1^+^/^−^ mice compared to WT mice. Transcriptomic analysis via RNA sequencing revealed upregulation of genes implicated in colitis and colorectal cancer (CRC) progression, such as *Anxa9*, *Atp2a1*, and *Hepacam2*, in ERAP1^+^/^−^ mice, regardless of sulfasalazine administration. These findings indicate that partial ERAP1 deficiency disrupts immune homeostasis and reduces the therapeutic efficacy of sulfasalazine, underscoring ERAP1 as a potential target for inflammatory bowel disease intervention.

DSS functions as a luminal toxin that disrupts the integrity of the colonic mucosal barrier. This disruption exacerbates both local and systemic inflammation and increases intestinal permeability (9). In this study, DSS-treated ERAP1 WT and ERAP1^+^/^−^ mice exhibited severe intestinal inflammation, accompanied by a marked reduction in colon length. DSS treatment also induced substantial weight loss, with WT mice losing approximately 20% and ERAP1^+^/^−^ mice approximately 30% of their initial body weight. Sulfasalazine treatment significantly alleviated intestinal inflammation and partially restored colon length in ERAP1 WT mice. However, this therapeutic effect was not observed in ERAP1^+^/^−^ mice, suggesting that ERAP1 may play a critical role in protecting against colonic injury and modulating the anti-inflammatory efficacy of sulfasalazine. Although ERAP1 is not directly involved in sulfasalazine metabolism, which primarily depends on colonic microbial activity (53), partial ERAP1 deficiency likely influences downstream immune modulation. Altered immune cell responses, dysregulated gene expression, and impaired antigen presentation collectively contribute to the reduced anti-inflammatory effects of sulfasalazine.

Genetic polymorphisms in ERAP1, leading to altered enzymatic activity, have been implicated in the pathogenesis of several autoimmune diseases, including IBD (24, 54). ERAP1 knockout mice (ERAP1^−^/^−^) show increased susceptibility to DSS-induced colitis, potentially driven by alterations in the gut microbiota (26), although the precise molecular mechanisms remain poorly understood. It is hypothesized that ERAP1 dysfunction compromises both innate and adaptive immune responses, thereby contributing to the onset and progression of autoimmune conditions (55). In our study, we used ERAP1^+^/^−^ mice to mimic the partial loss-of-function phenotype observed in human ERAP1 polymorphisms associated with immune dysregulation and autoinflammatory diseases (24). Unlike complete knockout models, which can cause broad immune alterations and trigger compensatory mechanisms (56), the haploinsufficient model enables investigation of more subtle and physiologically relevant changes in immune responses during colitis. This approach offers a clearer link to human pathophysiology, where complete loss-of-function mutations are uncommon.

Neutrophils are central effectors in UC, contributing to mucosal damage through the release of proteases, myeloperoxidase, NETs, and ROS. These factors disrupt the epithelial barrier, promote crypt abscess formation, and are implicated in the pathogenesis of colitis-associated colorectal cancer (13, 57). ERAP1 has been shown to regulate key aspects of neutrophil biology, including recruitment, activation, and cytokine production. Consequently, ERAP1 deficiency or dysregulation may enhance neutrophil-driven inflammation and contribute to disease progression (34, 58).

In our study, neutrophils were identified based on Ly6G^+^CD11b^+^ expression. ERAP1^+^/^−^ mice with DSS-induced colitis exhibited a significant increase in circulating Ly6G^+^CD11b^+^ neutrophils compared to ERAP1^+^/^−^ healthy controls, even following sulfasalazine treatment. This suggests that sulfasalazine fails to effectively suppress neutrophilic inflammation in the context of ERAP1 haploinsufficiency. Similarly, ERAP1 WT colitis mice also showed elevated levels of Ly6G^+^CD11b^+^ neutrophils compared to healthy WT controls. Notably, under colitis conditions, ERAP1^+^/^−^ mice exhibited a 70% increase in Ly6G^+^CD11b^+^ neutrophils in IELs relative to ERAP1 WT mice, indicating that partial ERAP1 deficiency may enhanced neutrophil infiltration in the gut mucosa during colitis. These findings highlight the critical role of ERAP1 in modulating neutrophil-mediated immune responses and suggest that ERAP1 haploinsufficiency may compromise the therapeutic efficacy of anti-inflammatory agents such as sulfasalazine in colitis. Further studies evaluating activation-specific markers (e.g., CD66b, CD177) or neutrophil functions (e.g., ROS production, NET formation) are warranted to fully characterize their inflammatory role.

Dendritic cells (DCs) serve as a critical bridge between the innate and adaptive immune systems. Their immunogenic functions depend on the expression of co-stimulatory molecules such as CD40, CD80, CD83, and CD86 (59). These molecules are essential for effective antigen presentation, T cell activation, and immune regulation, all of which are implicated in the pathogenesis of IBD (60, 61). Among them, CD40–CD40L interactions are particularly associated with oxidative stress and the modulation of immune signaling pathways relevant to IBD development (62). In pediatric IBD patients, increased CD40^+^ and CD80^+^ cell populations have been observed in the colonic mucosa, suggesting heightened immune activation (62). Similarly, elevated numbers of CD83^+^ cells have been reported in the inflamed colonic tissues of colitis mice and IBD patients (63, 64), and CD11c^+^ cells are also upregulated in the inflamed mucosa and lamina propria of colitis models (65).

Our findings demonstrate that partial loss of ERAP1 expression significantly influences these immune populations. Specifically, we observed markedly increased frequencies of CD40^+^ and CD80^+^ cells in the PBLs of DSS-treated ERAP1^+^/^−^ mice, including those receiving sulfasalazine, compared with ERAP1^+^/^−^ healthy controls. Furthermore, ERAP1^+^/^−^ colitis mice exhibited higher levels of CD40^+^, CD80^+^, and CD83^+^ cells compared to WT colitis mice. Notably, CD40^+^ and CD11c^+^ cells were further elevated in sulfasalazine-treated ERAP1^+^/^−^ colitis mice than in WT counterparts, indicating an altered immune response and potential dysregulation in antigen presentation under conditions of ERAP1 haploinsufficiency. Additionally, CD11c^+^ cells were significantly increased in sulfasalazine-treated ERAP1^+^/^−^ mice relative to their healthy controls. In contrast, sulfasalazine reduced CD11c^+^ cell expression in WT colitis mice, suggesting a possible restorative immune effect in WT, but not in ERAP1^+^/^−^ conditions. Interestingly, reduced CD86 expression on DCs has been noted in IBD patients (66). Concordantly, our study revealed a marked reduction in CD86^+^ cell frequency in PBLs of ERAP1^+^/^−^ colitis mice, which was further diminished after sulfasalazine treatment. CD86^+^ cell levels were also 31% lower in IELs of ERAP1^+^/^−^ mice compared to WT controls under normal conditions. Collectively, these results underscore the role of ERAP1 in regulating DC-mediated immune responses and suggest that ERAP1 insufficiency may contribute to impaired immunoregulation in IBD.

T cell infiltration into the intestinal mucosa is a hallmark of IBD, perpetuating chronic inflammation through the disruption of immune homeostasis (67–69). CD4^+^ and CD8^+^ T cells represent the T lymphocyte subsets. ERAP1 is critical for antigenic peptide trimming, a prerequisite for optimal MHC class I presentation, and the activation of CD8^+^ T and NK cells (70). Proper interaction between MHC molecules and lymphocytes is crucial for effective immune surveillance. The proportion of CD4^+^ T cells in the mesenteric lymph nodes (mLN) was markedly reduced in mice treated with DSS during both the acute and chronic phases of colitis (71). In addition, type 1 regulatory T (Tr1) cells—a CD4^+^ T cell subset—are central to maintaining peripheral tolerance and controlling tissue inflammation (72). Reduced Tr1 populations have been observed in ERAP1^−^/^−^ mice (26), implying a link between ERAP1 expression and immune tolerance mechanisms.

Our data showed a decrease in CD4^+^ T cells in the PBLs of DSS-treated ERAP1^+^/^−^ mice compared to their healthy counterparts. WT colitis mice also exhibited reduced CD4^+^ T cell frequencies in IELs, although this was partially restored with sulfasalazine treatment. No significant difference in CD4^+^ T cell levels was observed between healthy WT and ERAP1^+^/^−^ mice, suggesting that colitis-induced changes are specifically affected by ERAP1 haploinsufficiency.

In addition, the dysregulation of CD8^+^ T cells and NK1.1^+^ cells has been implicated in colitis pathogenesis, with both protective and pathogenic roles reported (18, 71, 73, 74). Previous studies showed that ERAP1 deficiency impairs CD8^+^ T cell cytotoxicity and reduces surface expression of the activating receptor NKG2D on NK cells (75, 76). Consistent with this, our study found that CD8^+^NK1.1^+^ cell frequencies were significantly decreased in WT colitis mice compared to WT controls, but sulfasalazine treatment led to a 63% increase in these cells, suggesting an immunoregulatory role. In contrast, sulfasalazine-treated ERAP1^+^/^−^ colitis mice exhibited a reduction in both NK1.1^+^ and CD8^+^NK1.1^+^ cells compared to healthy ERAP1^+^/^−^ controls. CD8^+^ T cells were also decreased in the PBLs of ERAP1^+^/^−^ colitis mice relative to WT colitis mice, and NK1.1^+^ cell frequencies were significantly lower in the IELs of ERAP1^+^/^−^ colitis mice than WT colitis mice after sulfasalazine administration. These findings suggest that ERAP1 expression modulates NK1.1^+^CD8^+^ cell dynamics in colitis, and further mechanistic investigations are required.

To further explore molecular differences, we performed transcriptomic analysis and identified 428 DEGs between ERAP1 WT and ERAP1^+^/^−^ mice under steady-state conditions—111 upregulated (26%) and 317 downregulated (74%) in ERAP1^+^/^−^ mice. Several DEGs with known relevance to colitis and CRC were prioritized. Patients with UC are at increased risk for CRC (77, 78), and genes such as *Annexin A9* (*Anxa9*) and *Atp2a1* have been identified as poor prognostic markers in CRC and potential mediators of IBD progression (79, 80). *Anxa9* has been implicated in CRC via modulation of Wnt signaling (45), while *Atp2a1* may contribute by limiting CD8^+^ T cell infiltration (80). Although no significant differences were found in *Anxa9* and *Atp2a1* expression between WT and ERAP1^+^/^−^ mice under normal conditions, their expression was significantly elevated in ERAP1^+^/^−^ colitis mice compared to WT colitis mice following sulfasalazine treatment, implicating them in ERAP1-associated colitis pathogenesis and therapeutic response.

Additionally, Hepacam2, a member of the immunoglobulin superfamily involved in immune regulation and mitotic control, was upregulated by 53% in ERAP1^+^/^−^ mice under steady-state conditions. *Hepacam2* expression is positively correlated with immune cell infiltration, checkpoint regulation, genomic instability, and drug sensitivity in CRC (46, 49, 79). *Hepacam2* expression was further increased in ERAP1^+^/^−^ colitis mice following sulfasalazine treatment, reinforcing its potential as a biomarker for UC subtypes and ERAP1-related immune modulation.

Genes involved in epithelial repair were also examined. Gadd45β plays a protective role by regulating TGF-β signaling to promote epithelial repair, and Gadd45β-deficient mice exhibit markedly increased susceptibility to DSS-induced colitis and mortality (50). Similarly, Reg3b-deficient mice develop more severe colitis, with elevated colonic IL - 6 and the repair marker Ym1 compared with wild-type controls after DSS treatment (51). SPINK5, whose expression is reduced at both mRNA and protein levels in esophageal cancer and associated with tumor progression, has been suggested as a potential biomarker (81), although its role in colitis and CRC remains unclear. No significant differences in *Gadd45β*, *Reg3b*, or *Spink5* expression were observed in our study, their established functions in epithelial repair and inflammation suggest possible mechanisms through which ERAP1 activity could influence mucosal healing and cancer risk.

Taken together, these findings suggest that ERAP1 regulates disease susceptibility and therapeutic response at least in part through transcriptional control of genes involved in epithelial integrity, immune cell infiltration, and repair pathways. Further studies are warranted to determine whether ERAP1 directly regulates genes such as *Anxa9*, *Atp2a1*, and *Hepacam2*, and to elucidate how these pathways collectively modulate epithelial barrier function, immune responses, and therapeutic outcomes in UC.

Despite these novel insights, several limitations must be acknowledged. First, although ERAP1^+^/^−^ mice showed heightened colitis severity and altered immune cell profiles, the precise molecular mechanisms by which partial ERAP1 deficiency influences specific immune pathways (e.g., neutrophil activation, DC maturation, CD4^+^ T cell function) remain unclear. Second, sulfasalazine was less effective in ERAP1^+^/^−^ mice, suggesting potential interactions between ERAP1 and the drug that need further study. In addition, although we used a standard sulfasalazine dose (50 mg/kg/day), its pharmacokinetics have not been tested in ERAP1^+^/^−^ mice, so differences in drug absorption or metabolism cannot be ruled out. Lastly, our flow cytometry analyses focused on selected immune subsets; broader profiling, including Tregs and innate lymphoid cells, may offer a more comprehensive view of the immune landscape in ERAP1-associated colitis.

## 5 Conclusion

This study investigated the immunomodulatory role of ERAP1 in the context of DSS-induced colitis, focusing on immune cell dynamics, gene expression profiles, and therapeutic responses to sulfasalazine. ERAP1^+^/^−^ colitis mice showed mildly increased susceptibility to colitis, along with distinct changes in immune cell infiltration and gene expression profiles. Despite sulfasalazine’s well-established anti-inflammatory effects, it failed to alleviate symptoms in ERAP1^+^/^−^ mice. Transcriptomic analysis identified 428 differentially expressed genes between ERAP1^+^/^−^ and WT colons, among which 28 were associated with colitis or colorectal cancer. Notably, the expression of *Anxa9*, *Atp2a1*, and *Hepacam2* was significantly elevated in ERAP1^+^/^−^ mice following sulfasalazine treatment, as confirmed by RT-qPCR, indicating a potential reduction in drug efficacy due to ERAP1 haploinsufficiency. These findings suggest that ERAP1 plays a crucial role in regulating intestinal immune responses and shaping therapeutic outcomes in colitis. Further mechanistic studies are warranted to elucidate how ERAP1 modulates disease progression and to identify novel ERAP1-related therapeutic targets for the treatment of inflammatory bowel disease.

## Data Availability

The data presented in the study are deposited in the NCBI Gene Expression Omnibus (GEO) repository and can be accessed under accession number GSE299626.

## Supplementary material

Supplementary Figure 1
**(A, B)** Gating strategy for cells is outlined in black. **(C)** Frequencies of Ly6G+CD11b+ neutrophils in ERAP WT and ERAP^+^/^−^ mice. **(D)** Frequencies of CD8+NK1.1+ cells in ERAP WT and ERAP^+^/^−^ mice. Data are presented as mean ± SD (n = 4 – 6 per group).

Supplementary Figure 2 Validation of RNA-seq results by RT-qPCR. Relative mRNA expression levels of three selected transcripts, **(A)** *Gadd45b*, **(B)** *Reg3β*, and **(C)** *Spink5*, were measured in spleen tissue from ERAP1 WT and ERAP1^+^/^−^ mice. Data are presented as mean ± SD (n = 4 – 6 per group).

Supplementary Figure 3 RT-qPCR analyzed mRNA levels of Foxp3 and IL - 17 following DSS-induced colitis and sulfasalazine treatment. **(A)** Expression of *Foxp3*, and **(B)** *IL-17* in splenic tissue from ERAP WT and ERAP1^+^/^−^ mice. Data are shown as mean ± SD (n = 4 – 6 per group).

## References

[1] Du L Ha C. Epidemiology and pathogenesis of ulcerative colitis. Gastroenterol Clin North Am. (2020) 49:643–54. doi: 10.1016/j.gtc.2020.07.005, PMID: 33121686

[2] Segal JP LeBlanc JF Hart AL. Ulcerative colitis: an update. Clin Med (Lond). (2021) 21:135–9. doi: 10.7861/clinmed.2021-0080, PMID: 33762374 PMC8002778

[3] DeRoche TC Xiao SY Liu X. Histological evaluation in ulcerative colitis. Gastroenterol Rep (Oxf). (2014) 2:178–92. doi: 10.1093/gastro/gou031, PMID: 24942757 PMC4124271

[4] Xu M Kong Y Chen N Peng W Zi R Jiang M. Identification of immune-related gene signature and prediction of cerna network in active ulcerative colitis. Front Immunol. (2022) 13:855645. doi: 10.3389/fimmu.2022.855645, PMID: 35392084 PMC8980722

[5] Guo XY Liu XJ Hao JY. Gut microbiota in ulcerative colitis: insights on pathogenesis and treatment. J Dig Dis. (2020) 21:147–59. doi: 10.1111/1751-2980.12849, PMID: 32040250

[6] Kałużna A Olczyk P Komosińska-Vassev K. The role of innate and adaptive immune cells in the pathogenesis and development of the inflammatory response in ulcerative colitis. J Clin Med. (2022) 11:400. doi: 10.3390/jcm11020400, PMID: 35054093 PMC8780689

[7] Jostins L Ripke S Weersma RK Duerr RH McGovern DP Hui KY. Host-microbe interactions have shaped the genetic architecture of inflammatory bowel disease. Nature. (2012) 491:119–24. doi: 10.1038/nature11582, PMID: 23128233 PMC3491803

[8] Armuzzi A Liguori G. Quality of life in patients with moderate to severe ulcerative colitis and the impact of treatment: A narrative review. Dig Liver Dis. (2021) 53:803–8. doi: 10.1016/j.dld.2021.03.002, PMID: 33744172

[9] Eichele DD Kharbanda KK. Dextran sodium sulfate colitis murine model: an indispensable tool for advancing our understanding of inflammatory bowel diseases pathogenesis. World J Gastroenterol. (2017) 23:6016–29. doi: 10.3748/wjg.v23.i33.6016, PMID: 28970718 PMC5597494

[10] Kiesler P Fuss IJ Strober W. Experimental models of inflammatory bowel diseases. Cell Mol Gastroenterol Hepatol. (2015) 1:154–70. doi: 10.1016/j.jcmgh.2015.01.006, PMID: 26000334 PMC4435576

[11] Chassaing B Aitken JD Malleshappa M Vijay-Kumar M. Dextran sulfate sodium (Dss)-induced colitis in mice. Curr Protoc Immunol. (2014) 104:15.25.1–15.25.14. doi: 10.1002/0471142735.im1525s104, PMID: 24510619 PMC3980572

[12] Wirtz S Popp V Kindermann M Gerlach K Weigmann B Fichtner-Feigl S. Chemically induced mouse models of acute and chronic intestinal inflammation. Nat Protoc. (2017) 12:1295–309. doi: 10.1038/nprot.2017.044, PMID: 28569761

[13] Zhang C Zhang J Zhang Y Song Z Bian J Yi H. Identifying neutrophil-associated subtypes in ulcerative colitis and confirming neutrophils promote colitis-associated colorectal cancer. Front Immunol. (2023) 14:1095098. doi: 10.3389/fimmu.2023.1095098, PMID: 36845139 PMC9950623

[14] Baumgart DC Metzke D Guckelberger O Pascher A Grötzinger C Przesdzing I. Aberrant plasmacytoid dendritic cell distribution and function in patients with Crohn’s disease and ulcerative colitis. Clin Exp Immunol. (2011) 166:46–54. doi: 10.1111/j.1365-2249.2011.04439.x, PMID: 21762123 PMC3193918

[15] Roberts-Thomson IC Fon J Uylaki W Cummins AG Barry S. Cells, cytokines and inflammatory bowel disease: A clinical perspective. Expert Rev Gastroenterol Hepatol. (2011) 5:703–16. doi: 10.1586/egh.11.74, PMID: 22017698

[16] Funderburg NT Stubblefield Park SR Sung HC Hardy G Clagett B Ignatz-Hoover J. Circulating Cd4(+) and Cd8(+) T cells are activated in inflammatory bowel disease and are associated with plasma markers of inflammation. Immunology. (2013) 140:87–97. doi: 10.1111/imm.12114, PMID: 23600521 PMC3809709

[17] Rabe H Malmquist M Barkman C Östman S Gjertsson I Saalman R. Distinct patterns of naive, activated and memory T and B cells in blood of patients with ulcerative colitis or Crohn’s disease. Clin Exp Immunol. (2019) 197:111–29. doi: 10.1111/cei.13294, PMID: 30883691 PMC6591150

[18] Hall LJ Murphy CT Quinlan A Hurley G Shanahan F Nally K. Natural killer cells protect mice from Dss-induced colitis by regulating neutrophil function via the Nkg2a receptor. Mucosal Immunol. (2013) 6:1016–26. doi: 10.1038/mi.2012.140, PMID: 23340823

[19] Nakase H Sato N Mizuno N Ikawa Y. The influence of cytokines on the complex pathology of ulcerative colitis. Autoimmun Rev. (2022) 21:103017. doi: 10.1016/j.autrev.2021.103017, PMID: 34902606

[20] Singh UP Singh NP Murphy EA Price RL Fayad R Nagarkatti M. Chemokine and cytokine levels in inflammatory bowel disease patients. Cytokine. (2016) 77:44–9. doi: 10.1016/j.cyto.2015.10.008, PMID: 26520877 PMC4666758

[21] Liu S Lu J Wu J Feng D Wang Y Su X. Structural and biochemical insights into the association between erap1 polymorphism and autoimmune diseases. Biochem Biophys Res Commun. (2022) 632:189–94. doi: 10.1016/j.bbrc.2022.09.086, PMID: 36228519

[22] Chen H Li L Weimershaus M Evnouchidou I van Endert P Bouvier M. Erap1-Erap2 dimers trim Mhc I-bound precursor peptides; implications for understanding peptide editing. Sci Rep. (2016) 6:28902. doi: 10.1038/srep28902, PMID: 27514473 PMC4981824

[23] Kochan G Krojer T Harvey D Fischer R Chen L Vollmar M. Crystal structures of the endoplasmic reticulum aminopeptidase-1 (Erap1) reveal the molecular basis for N-terminal peptide trimming. Proc Natl Acad Sci U.S.A. (2011) 108:7745–50. doi: 10.1073/pnas.1101262108, PMID: 21508329 PMC3093473

[24] Reeves E James E. The role of polymorphic Erap1 in autoinflammatory disease. Biosci Rep. (2018) 38:BSR20171503. doi: 10.1042/bsr20171503, PMID: 30054427 PMC6131210

[25] Blake MK O’Connell P Pepelyayeva Y Godbehere S Aldhamen YA Amalfitano A. Erap1 is a critical regulator of inflammasome-mediated proinflammatory and Er stress responses. BMC Immunol. (2022) 23:9. doi: 10.1186/s12865-022-00481-9, PMID: 35246034 PMC8895631

[26] Pepelyayeva Y Rastall DPW Aldhamen YA O’Connell P Raehtz S Alyaqoub FS. Erap1 deficient mice have reduced type 1 regulatory T cells and develop skeletal and intestinal features of ankylosing spondylitis. Sci Rep. (2018) 8:12464. doi: 10.1038/s41598-018-30159-5, PMID: 30127455 PMC6102283

[27] Zhao C Yu Y Yin G Xu C Wang J Wang L. Sulfasalazine promotes ferroptosis through Akt-Erk1/2 and P53-Slc7a11 in rheumatoid arthritis. Inflammopharmacology. (2024) 32:1277–94. doi: 10.1007/s10787-024-01439-6, PMID: 38407703 PMC11006818

[28] Yousefi-Ahmadipour A Ebrahimi-Barough S Niknia S Allahverdi A Mirzahosseini-Pourranjbar A Tashakori M. Therapeutic effects of combination of platelet lysate and sulfasalazine administration in Tnbs-induced colitis in rat. BioMed Pharmacother. (2020) 125:109949. doi: 10.1016/j.biopha.2020.109949, PMID: 32058216

[29] Svartz M. The treatment of 124 cases of ulcerative colitis with salazopyrine and attempts of desensibilization in cases of hypersensitiveness to sulfa. Acta Med Scand. (1948) 131:465–72. doi: 10.1111/j.0954-6820.1948.tb12083.x, PMID: 18881171

[30] Peppercorn MA Goldman P. The role of intestinal bacteria in the metabolism of salicylazosulfapyridine. J Pharmacol Exp Ther. (1972) 181:555–62. doi: 10.1016/S0022-3565(25)29238-2, PMID: 4402374

[31] Lima SF Pires S Rupert A Oguntunmibi S Jin WB Marderstein A. The gut microbiome regulates the clinical efficacy of sulfasalazine therapy for Ibd-associated spondyloarthritis. Cell Rep Med. (2024) 5:101431. doi: 10.1016/j.xcrm.2024.101431, PMID: 38378002 PMC10982976

[32] Azad Khan AK Piris J Truelove SC. An experiment to determine the active therapeutic moiety of sulphasalazine. Lancet. (1977) 2:892–5. doi: 10.1016/s0140-6736(77)90831-5, PMID: 72239

[33] Nikfar S Rahimi R Rezaie A Abdollahi M. A meta-analysis of the efficacy of sulfasalazine in comparison with 5-aminosalicylates in the induction of improvement and maintenance of remission in patients with ulcerative colitis. Dig Dis Sci. (2009) 54:1157–70. doi: 10.1007/s10620-008-0481-x, PMID: 18770034

[34] Ryu HM Islam SMS Sayeed HM Babita R Seong JK Lee H. Characterization of immune responses associated with Erap-1 expression in Hsv-induced Behçet’s disease mouse model. Clin Immunol. (2023) 250:109305. doi: 10.1016/j.clim.2023.109305, PMID: 37003592

[35] Qiu X Zhang F Yang X Wu N Jiang W Li X. Changes in the composition of intestinal fungi and their role in mice with dextran sulfate sodium-induced colitis. Sci Rep. (2015) 5:10416. doi: 10.1038/srep10416, PMID: 26013555 PMC4445066

[36] Nguyen TL Vieira-Silva S Liston A Raes J. How informative is the mouse for human gut microbiota research? Dis Model Mech. (2015) 8:1–16. doi: 10.1242/dmm.017400, PMID: 25561744 PMC4283646

[37] Couter CJ Surana NK. Isolation and flow cytometric characterization of murine small intestinal lymphocytes. J Vis Exp. (2016) 111):54114. doi: 10.3791/54114, PMID: 27213538 PMC4942069

[38] Jauregui-Amezaga A Geerits A Das Y Lemmens B Sagaert X Bessissow T. A simplified geboes score for ulcerative colitis. J Crohns Colitis. (2017) 11:305–13. doi: 10.1093/ecco-jcc/jjw154, PMID: 27571771

[39] Andrews S. Fastqc: A quality control tool for high throughput sequence data. (2010) Available online at: https://www.bioinformatics.babraham.ac.uk/projects/fastqc/.

[40] Bushnell B. Bbmap: A Fast, Accurate, Splice-Aware Aligner. No. LBNL-7065E. Berkeley, CA: Ernest Orlando Lawrence Berkeley National Laboratory. (2014).

[41] Dobin A Davis CA Schlesinger F Drenkow J Zaleski C Jha S. Star: ultrafast universal rna-seq aligner. Bioinformatics. (2013) 29:15–21. doi: 10.1093/bioinformatics/bts635, PMID: 23104886 PMC3530905

[42] Putri GH Anders S Pyl PT Pimanda JE Zanini F. Analysing high-throughput sequencing data in python with Htseq 2.0. Bioinformatics. (2022) 38:2943–5. doi: 10.1093/bioinformatics/btac166, PMID: 35561197 PMC9113351

[43] Dalmer TRA Clugston RD. Gene ontology enrichment analysis of congenital diaphragmatic hernia-associated genes. Pediatr Res. (2019) 85:13–9. doi: 10.1038/s41390-018-0192-8, PMID: 30287891 PMC6760551

[44] DennisGJr.ShermanBTHosackDAYangJGaoWLaneHC. David: database for annotation, visualization, and integrated discovery. Genome Biol. (2003) 4:P3. doi: 10.1186/gb-2003-4-5-p3, PMID: 12734009

[45] Lu X Hu L Mao J Zhang S Cai Y Chen W. Annexin A9 promotes cell proliferation by regulating the wnt signaling pathway in colorectal cancer. Hum Cell. (2023) 36:1729–40. doi: 10.1007/s13577-023-00939-x, PMID: 37349657 PMC10390359

[46] Wang S Zhang L Li D Gou M. Comprehensive analysis and experimental validation of Hepacam2 as a potential prognosis biomarker and immunotherapy target in colorectal cancer. Curr Gene Ther. (2024) 25(4):518–31. doi: 10.2174/0115665232325395241018103006, PMID: 39492767

[47] Rubin DC Shaker A Levin MS. Chronic intestinal inflammation: inflammatory bowel disease and colitis-associated colon cancer. Front Immunol. (2012) 3:107. doi: 10.3389/fimmu.2012.00107, PMID: 22586430 PMC3347037

[48] Zou M Liang Q Zhang W Zhu Y Xu Y. Endoplasmic reticulum stress related genome-wide mendelian randomization identifies therapeutic genes for ulcerative colitis and Crohn’s disease. Front Genet. (2023) 14:1270085. doi: 10.3389/fgene.2023.1270085, PMID: 37860672 PMC10583552

[49] Xie Y Li J Tao Q Wu Y Liu Z Chen Y. Identification of subclusters and prognostic genes based on gls-associated molecular signature in ulcerative colitis. Sci Rep. (2024) 14:13102. doi: 10.1038/s41598-024-63891-2, PMID: 38849409 PMC11161595

[50] Hwang JH Kim TH Kim YH Noh JR Choi DH Kim KS. Gadd45β promotes regeneration after injury through Tgfβ-dependent restitution in experimental colitis. Exp Mol Med. (2019) 51:1–14. doi: 10.1038/s12276-019-0335-y, PMID: 31666502 PMC6821912

[51] Shindo R Katagiri T Komazawa-Sakon S Ohmuraya M Takeda W Nakagawa Y. Regenerating islet-derived protein (Reg)3β Plays a crucial role in attenuation of ileitis and colitis in mice. Biochem Biophys Rep. (2020) 21:100738. doi: 10.1016/j.bbrep.2020.100738, PMID: 32072024 PMC7016002

[52] Tang L Liu Y Tao H Feng W Ren C Shu Y. Combination of Youhua Kuijie prescription and sulfasalazine can alleviate experimental colitis via Il-6/Jak2/Stat3 pathway. Front Pharmacol. (2024) 15:1437503. doi: 10.3389/fphar.2024.1437503, PMID: 39318778 PMC11420560

[53] Zheng H Chen M Li Y Wang Y Wei L Liao Z. Modulation of gut microbiome composition and function in experimental colitis treated with sulfasalazine. Front Microbiol. (2017) 8:1703. doi: 10.3389/fmicb.2017.01703, PMID: 28936203 PMC5594074

[54] Castro-Santos P Moro-García MA Marcos-Fernández R Alonso-Arias R Díaz-Peña R. Erap1 and Hla-C interaction in inflammatory bowel disease in the Spanish population. Innate Immun. (2017) 23:476–81. doi: 10.1177/1753425917716527, PMID: 28651467

[55] Pepelyayeva Y Amalfitano A. The role of Erap1 in autoinflammation and autoimmunity. Hum Immunol. (2019) 80:302–9. doi: 10.1016/j.humimm.2019.02.013, PMID: 30817945

[56] Cifaldi L Romania P Lorenzi S Locatelli F Fruci D. Role of endoplasmic reticulum aminopeptidases in health and disease: from infection to cancer. Int J Mol Sci. (2012) 13:8338–52. doi: 10.3390/ijms13078338, PMID: 22942706 PMC3430237

[57] Pineton de Chambrun M Wechsler B Geri G Cacoub P Saadoun D. New insights into the pathogenesis of Behçet’s disease. Autoimmun Rev. (2012) 11:687–98. doi: 10.1016/j.autrev.2011.11.026, PMID: 22197900

[58] Saulle I Limanaqi F Garziano M Murno ML Artusa V Strizzi S. Impact of endoplasmic reticulum aminopeptidases 1 (Erap1) and 2 (Erap2) on neutrophil cellular functions. Front Cell Dev Biol. (2024) 12:1506216. doi: 10.3389/fcell.2024.1506216, PMID: 39839670 PMC11747162

[59] Carenza C Calcaterra F Oriolo F Di Vito C Ubezio M Della Porta MG. Costimulatory molecules and immune checkpoints are differentially expressed on different subsets of dendritic cells. Front Immunol. (2019) 10:1325. doi: 10.3389/fimmu.2019.01325, PMID: 31244860 PMC6579930

[60] Mellman I. Dendritic cells: master regulators of the immune response. Cancer Immunol Res. (2013) 1:145–9. doi: 10.1158/2326-6066.Cir-13-0102, PMID: 24777676

[61] He W Wang B Li Q Yao Q Jia X Song R. Aberrant expressions of co-stimulatory and co-inhibitory molecules in autoimmune diseases. Front Immunol. (2019) 10:261. doi: 10.3389/fimmu.2019.00261, PMID: 30842773 PMC6391512

[62] Senhaji N Kojok K Darif Y Fadainia C Zaid Y. The contribution of Cd40/Cd40l axis in inflammatory bowel disease: an update. Front Immunol. (2015) 6:529. doi: 10.3389/fimmu.2015.00529, PMID: 26528290 PMC4607859

[63] Eckhardt J Kreiser S Döbbeler M Nicolette C DeBenedette MA Tcherepanova IY. Soluble Cd83 ameliorates experimental colitis in mice. Mucosal Immunol. (2014) 7:1006–18. doi: 10.1038/mi.2013.119, PMID: 24424524

[64] Riaz B Islam SMS Ryu HM Sohn S. Cd83 regulates the immune responses in inflammatory disorders. Int J Mol Sci. (2023) 24:2831. doi: 10.3390/ijms24032831, PMID: 36769151 PMC9917562

[65] Krajina T Leithäuser F Möller P Trobonjaca Z Reimann J. Colonic lamina propria dendritic cells in mice with Cd4+ T cell-induced colitis. Eur J Immunol. (2003) 33:1073–83. doi: 10.1002/eji.200323518, PMID: 12672074

[66] Baumgart DC Metzke D Schmitz J Scheffold A Sturm A Wiedenmann B. Patients with active inflammatory bowel disease lack immature peripheral blood plasmacytoid and myeloid dendritic cells. Gut. (2005) 54:228–36. doi: 10.1136/gut.2004.040360, PMID: 15647187 PMC1774844

[67] Cao H Diao J Liu H Liu S Liu J Yuan J. The pathogenicity and synergistic action of Th1 and Th17 cells in inflammatory bowel diseases. Inflammation Bowel Dis. (2023) 29:818–29. doi: 10.1093/ibd/izac199, PMID: 36166586

[68] Gomez-Bris R Saez A Herrero-Fernandez B Rius C Sanchez-Martinez H Gonzalez-Granado JM. Cd4 T-cell subsets and the pathophysiology of inflammatory bowel disease. Int J Mol Sci. (2023) 24:2696. doi: 10.3390/ijms24032696, PMID: 36769019 PMC9916759

[69] Friedrich M Pohin M Powrie F. Cytokine networks in the pathophysiology of inflammatory bowel disease. Immunity. (2019) 50:992–1006. doi: 10.1016/j.immuni.2019.03.017, PMID: 30995511

[70] Seamon K Kurlak LO Warthan M Stratikos E Strauss JF 3rd Mistry HD. The differential expression of Erap1/Erap2 and immune cell activation in pre-eclampsia. Front Immunol. (2020) 11:396. doi: 10.3389/fimmu.2020.00396, PMID: 32210971 PMC7076169

[71] Hall LJ Faivre E Quinlan A Shanahan F Nally K Melgar S. Induction and activation of adaptive immune populations during acute and chronic phases of a murine model of experimental colitis. Dig Dis Sci. (2011) 56:79–89. doi: 10.1007/s10620-010-1240-3, PMID: 20467900

[72] Zhou JY Glendenning LM Cavanaugh JM McNeer SK Goodman WA Cobb BA. Intestinal tr1 cells confer protection against colitis in the absence of Foxp3+ Regulatory T cell-derived Il-10. Immunohorizons. (2023) 7:456–66. doi: 10.4049/immunohorizons.2200071, PMID: 37314833 PMC10580124

[73] Casalegno Garduño R Däbritz J. New insights on Cd8(+) T cells in inflammatory bowel disease and therapeutic approaches. Front Immunol. (2021) 12:738762. doi: 10.3389/fimmu.2021.738762, PMID: 34707610 PMC8542854

[74] Yoshioka K Ueno Y Tanaka S Nagai K Onitake T Hanaoka R. Role of natural killer T cells in the mouse colitis-associated colon cancer model. Scand J Immunol. (2012) 75:16–26. doi: 10.1111/j.1365-3083.2011.02607.x, PMID: 21815907

[75] Cifaldi L Romania P Falco M Lorenzi S Meazza R Petrini S. Erap1 regulates natural killer cell function by controlling the engagement of inhibitory receptors. Cancer Res. (2015) 75:824–34. doi: 10.1158/0008-5472.Can-14-1643, PMID: 25592150

[76] Rastall DPW Alyaquob FS O’Connell P Pepelyayeva Y Peters D Godbehere-Roosa S. Mice expressing human erap1 variants associated with ankylosing spondylitis have altered T-cell repertoires and nk cell functions, as well as increased in utero and perinatal mortality. Int Immunol. (2017) 29:277–89. doi: 10.1093/intimm/dxx035, PMID: 28814066 PMC5890900

[77] Rivera AP Flores Monar GV Islam H Puttagunta SM Islam R Kundu S. Ulcerative colitis-induced colorectal carcinoma: A deleterious concatenation. Cureus. (2022) 14:e22636. doi: 10.7759/cureus.22636, PMID: 35371788 PMC8959421

[78] Bopanna S Ananthakrishnan AN Kedia S Yajnik V Ahuja V. Risk of colorectal cancer in Asian patients with ulcerative colitis: A systematic review and meta-analysis. Lancet Gastroenterol Hepatol. (2017) 2:269–76. doi: 10.1016/s2468-1253(17)30004-3, PMID: 28404156 PMC5713894

[79] Ntunzwenimana JC Boucher G Paquette J Gosselin H Alikashani A Morin N. Functional screen of inflammatory bowel disease genes reveals key epithelial functions. Genome Med. (2021) 13:181. doi: 10.1186/s13073-021-00996-7, PMID: 34758847 PMC8582123

[80] Zhang G Shang H Liu B Wu G Wu D Wang L. Increased Atp2a1 predicts poor prognosis in patients with colorectal carcinoma. Front Genet. (2022) 13:661348. doi: 10.3389/fgene.2022.661348, PMID: 35783262 PMC9243465

[81] Zhao C Zhu H Tian Y Sun Y Zhang Z. Spink5 is a key regulator of eosinophil extracellular traps in head and neck squamous cell carcinoma. Discov Oncol. (2024) 15:627. doi: 10.1007/s12672-024-01513-z, PMID: 39508915 PMC11543977

