# Supplementary material for: Impact of ERAP1 downregulation on the pathogenesis of DSS-induced colitis and therapeutic response to sulfasalazine
Source: Front Immunol. 2025 Sep 5;16:1645678. doi: 10.3389/fimmu.2025.1645678 (PMC12446291; doi:10.3389/fimmu.2025.1645678)
Supplement: Supplementary Figure 1 — (A, B) Gating strategy for cells is outlined in black. (C) Frequencies of Ly6G+CD11b+ neutrophils in ERAP WT and ERAP+/− mice. (D) Frequencies of CD8+NK1.1+ cells in ERAP WT and ERAP+/− mice. Data are presented as mean ± SD (n = 4 – 6 per group). [file Table1.docx]

**Supplementary data**

**Supplementary figures**

**Figure S1**

**
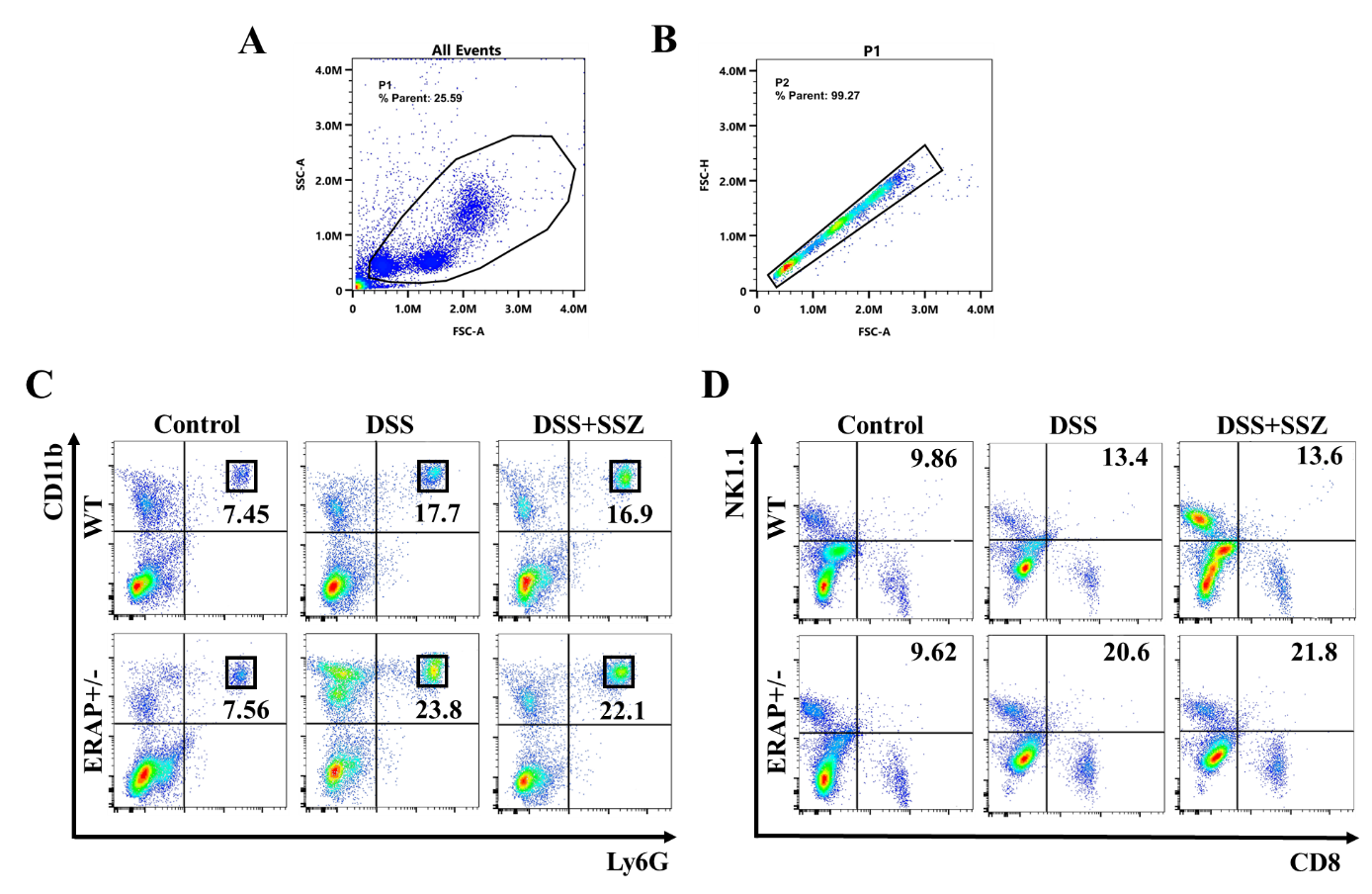
**

**Figure S2**


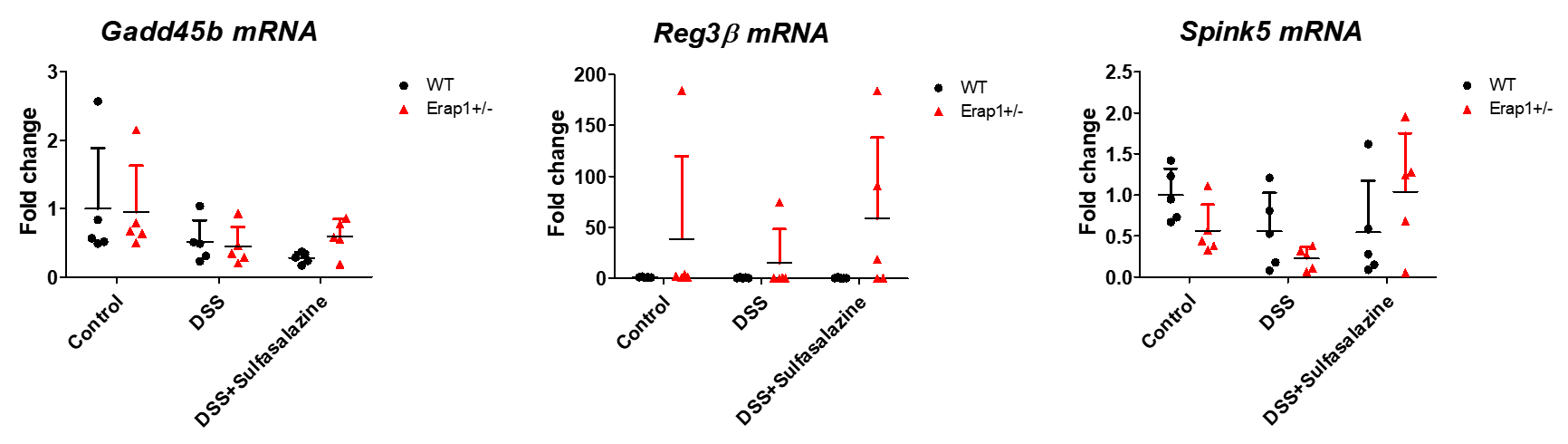


**Figure S3**

**Supplementary Table**

**Table S1.** List of primary antibodies used for flow cytometry

| Target | Clone | Fluorochrome | Manufacturer |
| --- | --- | --- | --- |
| CD40 | 1C10 | PerCP-eFluor 710 | eBioscience |
| CD80 | 16-10A1 | PE-Cyanine7 | eBioscience |
| CD83 | Michel-17 | eFluor 660 | eBioscience |
| CD86 | GL1 | FITC | eBioscience |
| CD4 | RM4-5 | APC | eBioscience |
| CD8 | 53-6.7 | PerCP-Cyanine5.5 | eBioscience |
| CD11c | N418 | PE | eBioscience |
| CD11b | M1/70 | PE | eBioscience |
| Ly6G | 1A8 | FITC | eBioscience |
| NK1.1 | PK136 | PE-Cyanine7 | BD Pharmigen |
